# Recent Advances in Fabrication of Non-Isocyanate Polyurethane-Based Composite Materials

**DOI:** 10.3390/ma14133497

**Published:** 2021-06-23

**Authors:** Piotr Stachak, Izabela Łukaszewska, Edyta Hebda, Krzysztof Pielichowski

**Affiliations:** Department of Chemistry and Technology of Polymers, Faculty of Chemical Engineering and Technology, Cracow University of Technology, Warszawska 24, 31-155 Cracow, Poland; izabela.lukaszewska@doktorant.pk.edu.pl (I.Ł.); edyta.hebda@pk.edu.pl (E.H.); kpielich@pk.edu.pl (K.P.)

**Keywords:** non-isocyanate polyurethanes, NIPU, copolymers, (nano)composites, hybrids, processing

## Abstract

Polyurethanes (PUs) are a significant group of polymeric materials that, due to their outstanding mechanical, chemical, and physical properties, are used in a wide range of applications. Conventionally, PUs are obtained in polyaddition reactions between diisocyanates and polyols. Due to the toxicity of isocyanate raw materials and their synthesis method utilizing phosgene, new cleaner synthetic routes for polyurethanes without using isocyanates have attracted increasing attention in recent years. Among different attempts to replace the conventional process, polyaddition of cyclic carbonates (CCs) and polyfunctional amines seems to be the most promising way to obtain non-isocyanate polyurethanes (NIPUs) or, more precisely, polyhydroxyurethanes (PHUs), while primary and secondary –OH groups are being formed alongside urethane linkages. Such an approach eliminates hazardous chemical compounds from the synthesis and leads to the fabrication of polymeric materials with unique and tunable properties. The main advantages include better chemical, mechanical, and thermal resistance, and the process itself is invulnerable to moisture, which is an essential technological feature. NIPUs can be modified via copolymerization or used as matrices to fabricate polymer composites with different additives, similar to their conventional counterparts. Hence, non-isocyanate polyurethanes are a new class of environmentally friendly polymeric materials. Many papers on the matter above have been published, including both original research and extensive reviews. However, they do not provide collected information on NIPU composites fabrication and processing. Hence, this review describes the latest progress in non-isocyanate polyurethane synthesis, modification, and finally processing. While focusing primarily on the carbonate/amine route, methods of obtaining NIPU are described, and their properties are presented. Ways of incorporating various compounds into NIPU matrices are characterized by the role of PHU materials in copolymeric materials or as an additive. Finally, diverse processing methods of non-isocyanate polyurethanes are presented, including electrospinning or 3D printing.

## 1. Introduction

Polyurethanes (PUs) are an important class of polymers obtained through the addition polymerization reaction of diisocyanates and diols. Due to their extraordinary mechanical, chemical, and physical properties (elasticity, biocompatibility, resistance to abrasion, tensile strength), PUs are used in a wide range of applications. Polyurethanes are used as rigid and flexible foams, namely, as coatings, glues, sealants, thermoplastic materials, elastomers, or thermosetting resins [1,2]. The applications include, but are not limited to, thermal insulation, textiles, bed mattresses and pillows, automotive parts, or scaffolds for tissue engineering.

**Scheme 1:**
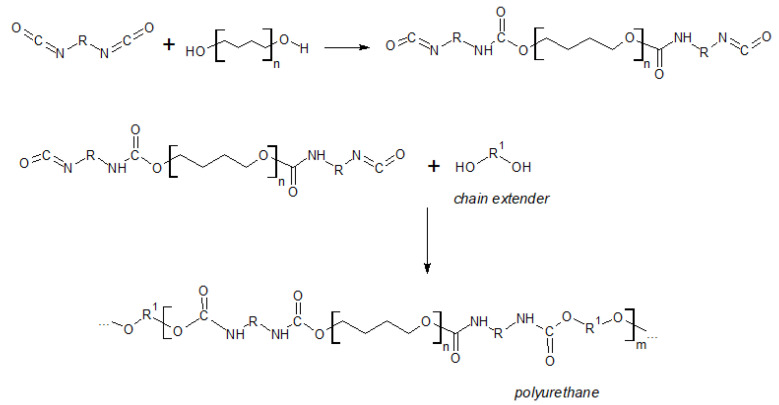
Synthesis of conventional polyurethane through a two-step method by reacting diisocyanate, polyol, and low molecular weight chain extender [1].

According to the method developed by Otto Bayer in the 1950s [1,3], PUs are obtained by a polyaddition reaction between aliphatic or aromatic isocyanate that contains at least two reactive groups and oligomeric polyol, possessing two or more reactive hydrogen atoms. Low molecular diols or diamines are used as chain extenders. Reactions are primarily carried out in the presence of the catalyst and a suitable solvent. Scheme 1 shows the conventional two-step PU synthesis—the first stage of the reaction consists of forming a prepolymer (oligomer end-capped with reactive moieties). A chain extender is added to the mixture in the second step, and finally, polyurethane is formed [1,2].

**Scheme 2:**
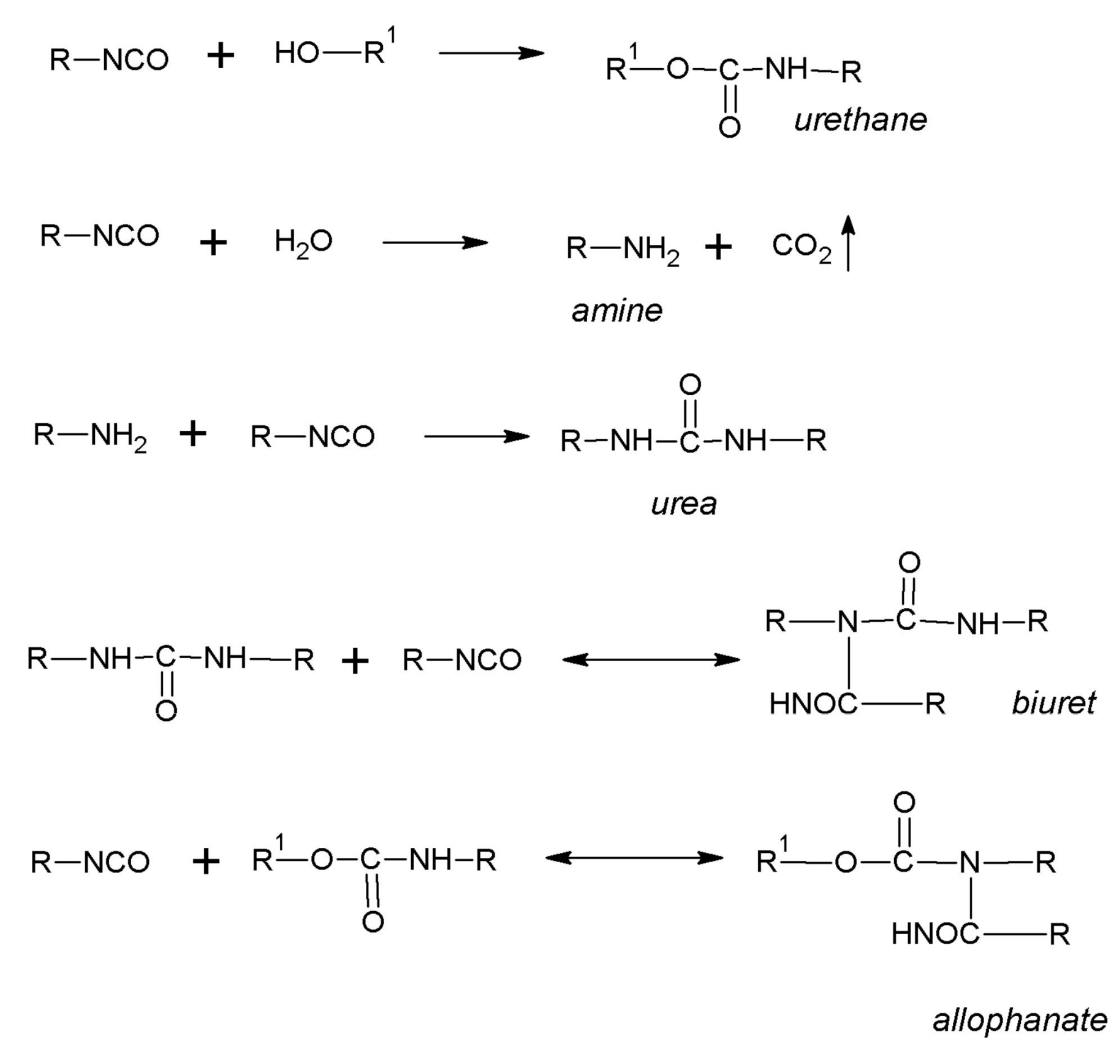
Urethane formation reaction and side reactions occurring during the synthesis of conventional polyurethanes [11].

However, side reactions accompany the main process, especially when moisture is present (Scheme 2). Simultaneously with the formation of urethane bonds, isocyanate groups may react with water, which leads to the formation of amine and carbon dioxide. This reaction is known as a foaming reaction, and while it is desired in the synthesis of foams, it is unwanted in obtaining polyurethane elastomers [4]. Formed amines further react with isocyanate groups, and urea moieties are formed [5,6].

The leading environmental issue of polyurethanes concerns the fact that isocyanate precursors show some toxicity [7]. The most frequently used isocyanates in the polyurethane industry are methylene diphenyl 4,4′-diisocyanate (MDI) and toluene diisocyanate (TDI). Although the toluene diisocyanate or methyl diisocyanates utilized in PU manufacturing are considered less of an irritant than methyl isocyanate (used in the production of pesticides) due to their lower volatilities, all abovementioned compounds are labeled as CMR (carcinogen, mutagen, and reprotoxic), which is a serious concern, especially for workers at polyurethane plants [8]. Lack of knowledge of potential health hazards concerning isocyanates may lead to more severe consequences. The main direct threats of human exposure to isocyanates involve occupational asthma, respiratory tract disorders, sensitization of respiratory tracts, or allergic reactions. As potent sensitizers, isocyanates stand as leading causes of occupational asthma around the world. Moreover, it is likely that sensitization by isocyanates through skin exposure may also be responsible for isocyanate-induced asthma [9,10].

**Scheme 3:**
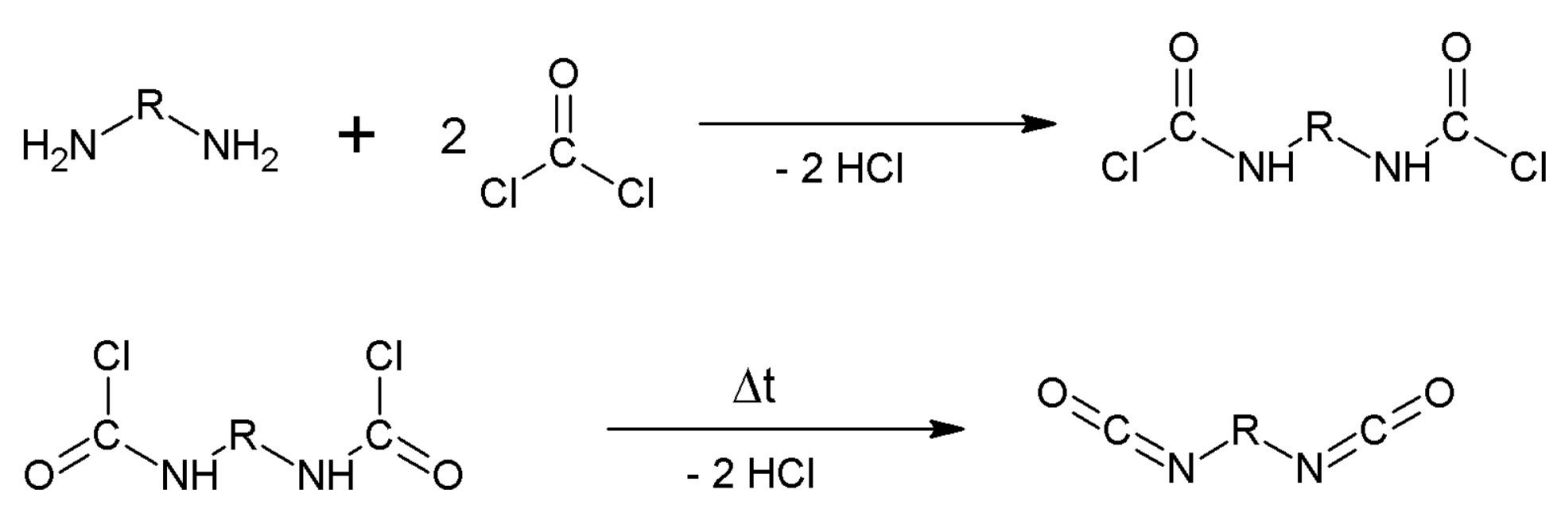
Synthesis of diisocyanate by phosgenation of primary amines [12].

Not only are isocyanates themselves hazardous but also the substrates used for their synthesis. On an industrial scale, isocyanates are obtained through the phosgene route (Scheme 3). Phosgene is a toxic, colorless gas that is acquired in the addition of chlorine to carbon monoxide [12].

The search for novel environmentally safe polyurethane materials with improved properties, synthesized without isocyanate components, has intensified in recent years. While synthesis of non-isocyanate polyurethanes has been extensively described, there is little information on the modification of NIPU matrices or on the processing methods. Original research papers and comprehensive reviews about NIPU synthesis and their properties can be found; however, only a few articles describe their processing or NIPU composites. Hence, the objective of this paper is to present and highlight the latest developments in the field of non-isocyanate polyurethane synthesis routes, as well as ways of their modification to obtain (nano)composites, with NIPU having different roles (matrices, additives, and copolymer components) in the final products, as well as their latter processing routes, including conventional and novel methods such as additive manufacturing.

## 2. NIPU Synthesis

**Scheme 4:**
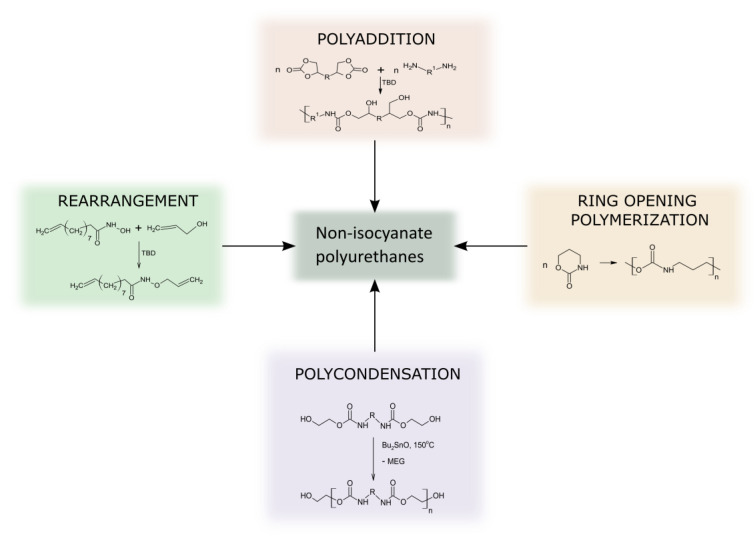
Possible pathways for non-isocyanate polyurethanes synthesis [12].

There are many ways to obtain non-isocyanate polyurethanes. The four main approaches consist of polyaddition, polycondensation, ring-opening polymerization, and rearrangement (Scheme 4).

Non-isocyanate PUs can be synthesized using cyclic carbonates and amines. While this is the most general approach, there are also some other methods involved. Polycondensation, ring-opening polymerization, and rearrangement reactions are described in the following paragraphs.

### 2.1. NIPU Synthesis via Polyaddition of Cyclic Carbonates and Amines

**Scheme 5:**
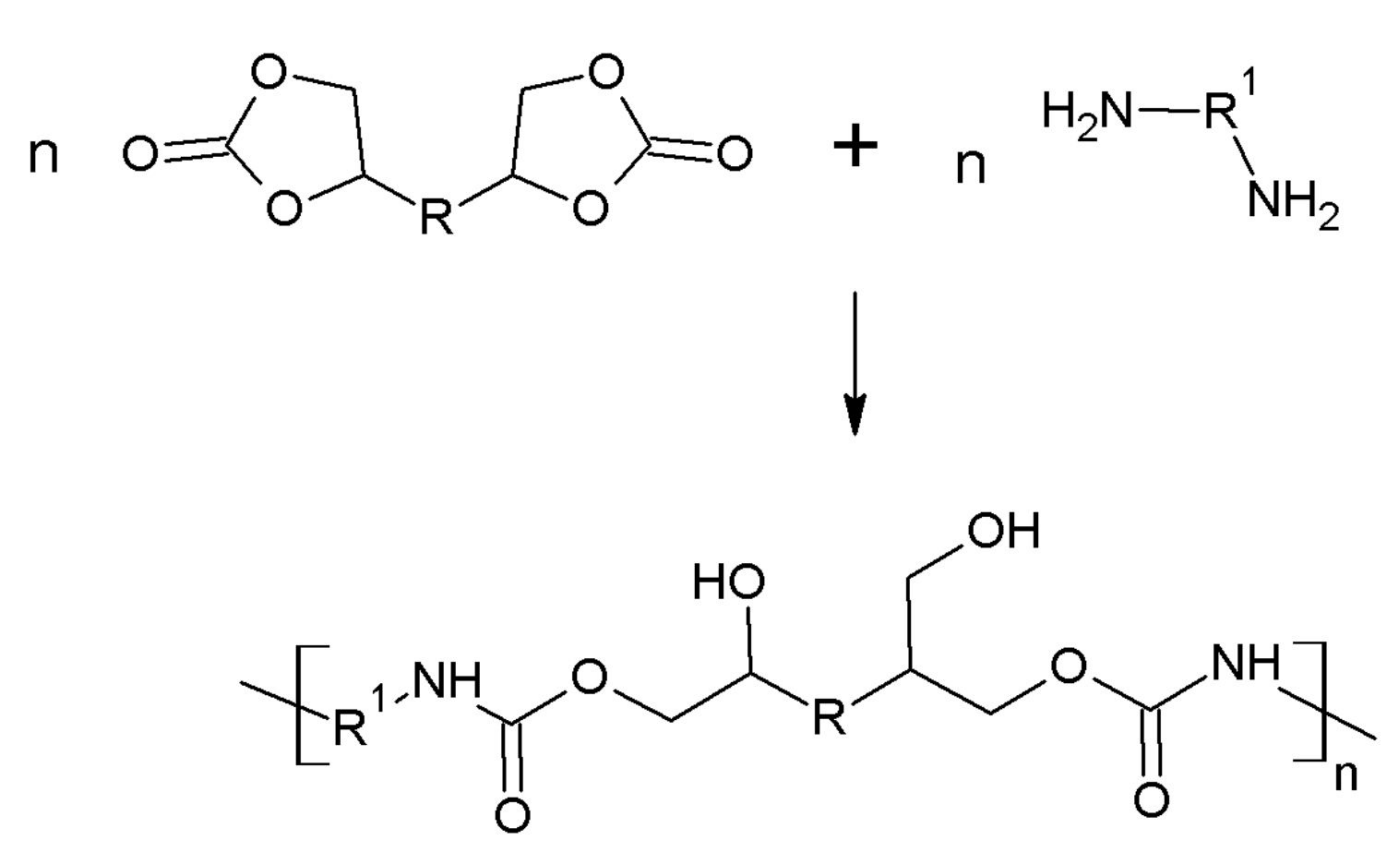
Synthesis of non-isocyanate polyhydroxyurethanes from cyclic carbonates and diamines [10].

The most direct way is the reaction of cyclic carbonate (CC) with the amine to yield non-isocyanate polyurethane (NIPU), on the condition that both reagents bear at least two of the respective reactive moieties (Scheme 5) [10,13].

With the formation of every urethane linkage, a primary or secondary hydroxyl group is also formed. Thus, NIPUs may alternatively be termed as polyhydroxyurethanes (PHU) [11,12].

Non-isocyanate polyurethane synthesis utilizing cyclic carbonates and amines has become popular as one of the emerging incentives. High solvency and high boiling points of cyclo-carbonates make them predominant over some other kinds of reagents. A wide range of possible reactions with various compounds, such as aliphatic or aromatic amines, alcohols, thiols, and carboxylic acids, opens possibilities for different synthetic routes. The most important qualities of cyclo-carbonates are biodegradability and low toxicity in comparison with isocyanates [10,14,15].

For the synthesis of NIPU, the conversion of epoxies using CO_2_ under the influence of catalysts is the leading pathway for obtaining five-membered cyclic carbonates. The abovementioned method is easy to carry out and yields high amounts of the main products without the formation of by-products or the necessity of using harmless reagents. Such qualities of CO_2_ as its abundance in nature, noncombustibility, nontoxicity, and being chemically inert and renewable make it a perfect candidate for environmentally friendly synthesis of cyclo-carbonates [5,12].

As an additional advantage, both cyclic carbonates and amines may be obtained from bio-based resources. For example, cyclic carbonates can be synthesized by chemical transformation of epoxidized oils derived from plants through glycerol carbonate-based intermediates or by utilizing reaction between CO_2_ and diols obtained from hydrogenolysis of biomass-derived compounds, such as cellulose, sorbitol, and glucose. Polyamines could be produced from fatty diacids. Such an environmentally friendly approach fortifies the safe application of CCs and amines in obtaining non-isocyanate polyurethanes [12,16].

An important category of NIPU as cross-linked materials requires at least one of the components to be polyfunctional, with at least three reactive groups in its structure. Two main strategies have been applied to fulfill this requirement—the first employs polyfunctional cyclic carbonates, whereby polyfunctional amines are used as curing agents in the second path [16,17,18].

**Scheme 6:**
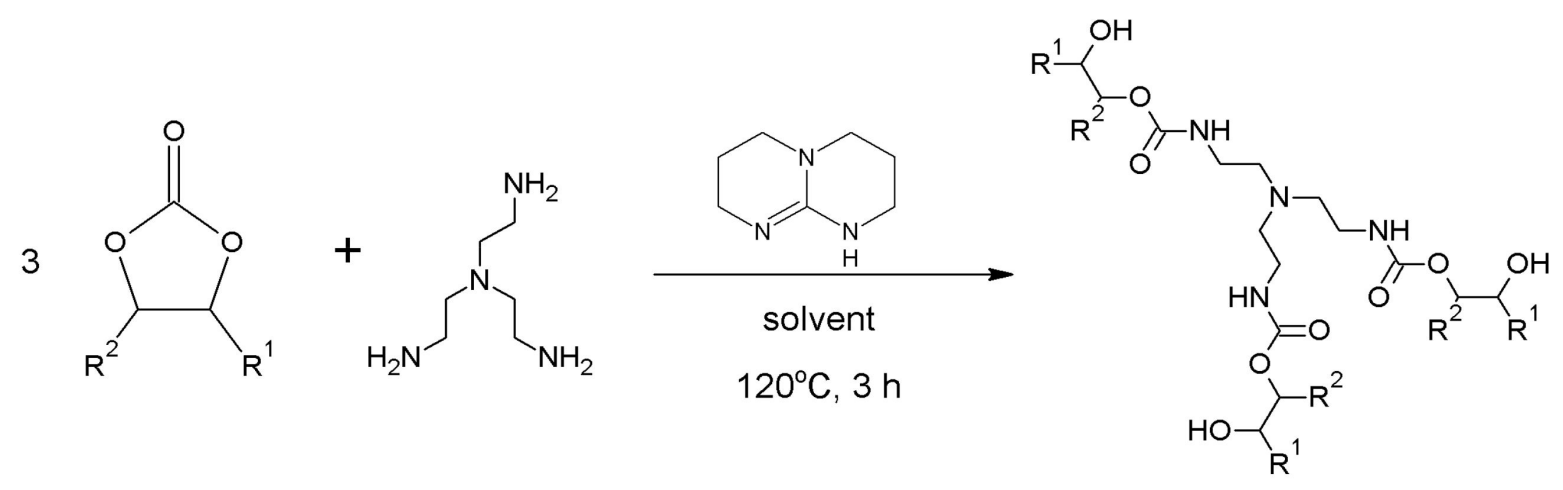
Crosslinking reaction of CSS with TAEA catalyzed by TBD [17].

The latter approach was utilized by Yu et al., who obtained bio-based resins by converting epoxidized soybean oil and sucrose soyate into carbonated soybean oil (CSO) and carbonated sucrose soyate (CSS), respectively, using CO_2_ under supercritical conditions, which is in accordance with green chemistry postulates to utilize renewable sources if possible. While focusing on developing formulations suitable for polyurethane coatings, various solvents and catalysts were tested during different process steps. Tris(2-aminoethyl)amine (TAEA) was used as a final curing agent with 1,5,7-triazabicyclo[4.4.0]dec-5-ene (TBD) in the role of a catalyst—Scheme 6 [17].

By selecting appropriate conditions (solvents, temperature, catalysts) to establish the best curing method, it was found that the choice of the catalyst is of great importance as it creates an equilibrium between vitrification and coating properties. Higher functionality of the carbonated sucrose soyate significantly influenced coating properties, cross-linking, and the glass transition temperatures of the coatings [17].

**Figure 1:**
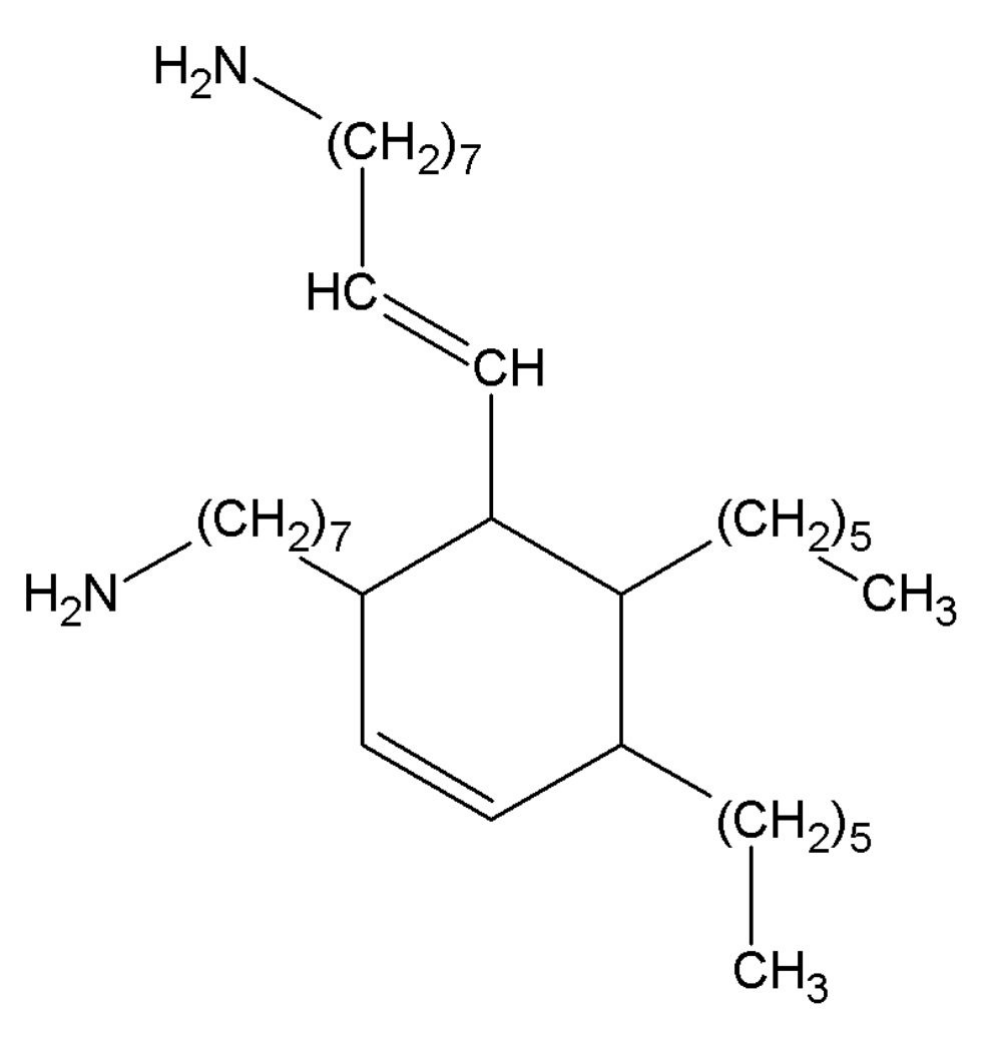
Molecular structure of diamine dimmer [16].

Carre et al., synthesized NIPUs from dimer-based diamines (DDA) and sebacic biscyclocarbonate (SB BisCC) with no solvent no catalyst included. The effect of the reaction conditions on the structures and properties of non-isocyanate polyurethanes with varying average amine functionalities (2.0 to 2.2) was described. As shown in Figure 1, DDAs are aliphatic compounds synthesized from dimer fatty acids [16].

**Scheme 7:**
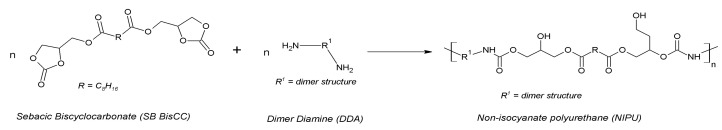
Reaction of SB BisCC with dimer-based diamine [16].

Two different DDAs were utilized as a standalone reactant or mixed to adjust a specific average amine functionality. The reaction lasted for two hours at a temperature of 75 °C under stirring and inert gas flow to avoid undesirable side reactions (Scheme 7) [16].

It turned out that the stoichiometric ratio happened to be optimal for the synthesis of NIPU with the highest molar masses (up to 22,000 g/mol). However, they were lower than conventional polyurethanes (ranging from 40,000 g/mol to 80,000 g/mol). Conversely, the dispersity of obtained NIPU ranging from 2.5 to 3.1 was higher than their conventional counterparts (1.6–2.2) [19,20]. Different properties of the obtained NIPU can be controlled by average amine functionalities of DDA and the cross-linking degree, which was confirmed by thermal and rheological analyses. Glass transition temperature of −23 °C to −14 °C was achieved for different NIPU formulations [16]. The range of *T_g_* for polyurethanes obtained via the isocyanate route varies greatly and depends on the compounds used. Nevertheless, it exists in the range of −75 °C up to 0 °C for soft segments and around 50 °C for hard segments [19,20].

**Scheme 8:**
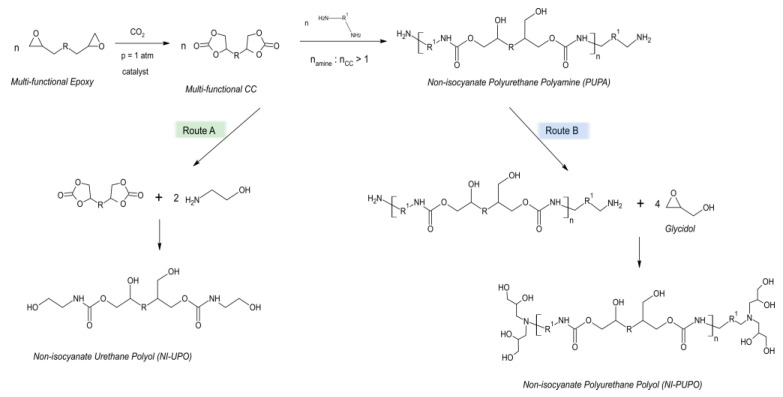
Schematic presentations of chemical routes used for the synthesis of (poly)urethane polyols [21].

Asemani and Mannari synthesized NIPU-polyols with varying hydroxyl content and Mw to be used later in thermosetting films. The first part of their work focused on synthesizing multi-functional cyclic carbonates (MFCCs) through the carbonation of oxirane precursors. Then, they obtained non-isocyanate polyurethane polyamine (PUPA) oligomers in the reaction between MFCCs with a 1.7 excess amount of isophorone diamine (IPDA). The second part of the research was focused on converting PUPAs to polyurethane polyols and their curing using a highly methylated, monomeric melamine crosslinker (HMMM)—Scheme 8 [21].

Utilizing three different epoxy compounds: 1,4-butanediol diglycidyl ether, polypropylene glycol diglycidyl ether, and propoxylated glycerin triglycidyl ether, MFCCs were obtained in the reaction of fixation of CO_2_ catalyzed by Methyltriphenylphosphonium iodide (Me.Ph.I) conducted under atmospheric pressure. Then, PUPA compounds were obtained in the reaction between cyclic carbonate compounds with a 1.7 excess of IPDA. The process was carried out in a solvent environment. Routes A and B (Scheme 8) show a further modification of PUPAs and cyclic carbonates to yield non-isocyanate polyurethane polyols, which were then cured to form coatings. Higher content of urethane moieties in the structure is beneficial in terms of adhesion to the substrate. The flexibility of the backbone of the polyol and the amount of crosslinker had an important effect on various properties, such as flexibility, impact resistance, and chemical resistance of obtained materials. As for thermal stability, prepared compositions achieved 50 wt% of decomposition in the temperature range of 370 °C to 424 °C [21], whereas conventional PUs start to decompose at a lower temperature of around 240 °C [22].

Wu et al., reacted diglycidyl ether of bisphenol-A and CO_2_ under elevated pressure, resulting in cyclic carbonate. Cyclic carbonate was prepared in a high-pressure stainless-steel reactor with tetrabutyl ammonium bromide (TBAB) acting as a catalyst. It was then dissolved in dimethylformamide (DMF), and IPDA was mixed in molar ratios from 10:1 to 10:6 (carbonate/primary amine). The process resulted in cyclic carbonate-terminated prepolymers. Further addition of polyethyleneimine (PEI), containing 6.71, 13.60, 21.50, 43.0, and 51.48 mol of primary amine groups, respectively, resulted in the final product. Finally, it was found that coat hardness and *T_g_* were increasing alongside IPDA content, contrary to the impact resistance and flexibility. The obtained films showed extraordinary hardness, impact strength, thermal stabilities, adhesion, flexibility, and chemical resistance to alkali and various solvents, such as toluene, xylene, and ethanol [23].

**Figure 2:**
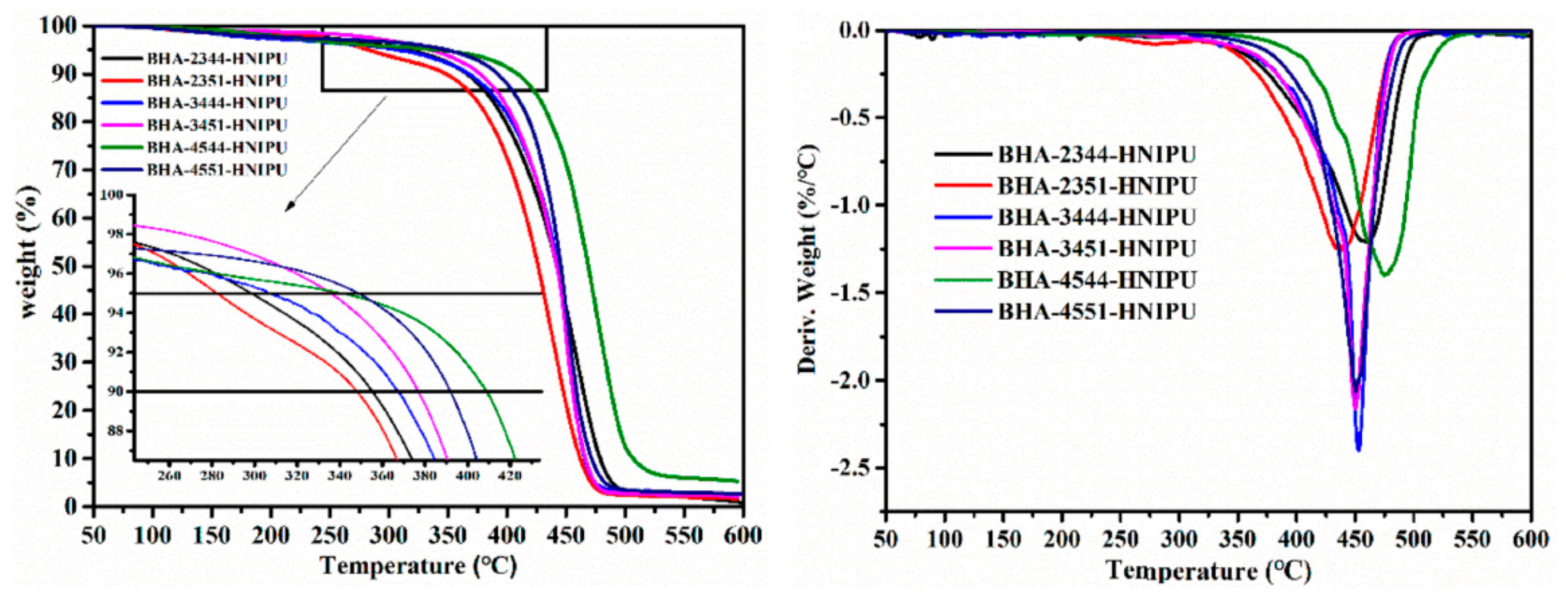
TGA (**left**) and DTG (**right**) curves of HNIPUs [24].

He et al., proposed an innovative type of solvent-free and catalyst-free process for obtaining hybrid non-isocyanate polyurethane (HNIPU). This approach is one of many ways to address the problem of using substances that are harmful to the environment. Solvent-free and catalyst-free synthesis is an eco-friendly way to obtain desired materials without stress on product purification and waste treatment. Four diamines—ethylenediamine (EDA), diethylenetriamine (DETA), triethylenetetramine (TETA), and tetraethylenepentamine (TEPA)—were used in the reaction with ethylene carbonate (EC) to obtain four bis(hydroxyethyloxycarbonylamino)alkanes (BHAs). To prepare different non-isocyanate polyurethane prepolymers, BHAs were reacted with dimer acid (DA–9-[(Z)-non-3-enyl]-10-octylnonadecanedioic acid). As the following step, epoxy resins were applied as the cross-linking agents to obtain HNIPUs by cross-linking and curing the prepolymers in the oven under a vacuum. Two bisphenol-A epoxy resins with different epoxy values (0.44 and 0.51) were selected and yielded 100% respective HNIPUs. Results from thermogravimetric analysis (TGA) and derivative thermogravimetry (DTG) showed that cross-linked HNIPUs had outstanding thermal stability, and the weight loss of 5% was achieved at ca. 350 °C for the best sample obtained (Figure 2). The obtained HNIPU show good thermal stability, so they can be considered heat-resistant coatings [24].

Ke et al., aimed at improving the performance of hybrid non-isocyanate polyurethanes by introducing methanol as a solvent for the reaction and utilizing different methods of synthesis: “all-in method”, “reverse method”, and “forward method”. In the all-in method, poly(propylene oxide) bis-(five-membered) cyclic carbonate (PPO-5CC) was directly mixed with 1,2-ethylenediamine (EDA) in methanol in the presence of triethylenediamine (TEDA) as a catalyst. The mixture was heated to 50 °C and stirred for 4 h, then methanol was removed by using negative-pressure filtration at 70 °C for 8 h. To discover the effect of the used solvent on the molar mass of obtained non-isocyanate polyurethane prepolymer, the ring-opening reaction was also conducted at 90 °C in the absence of methanol by the all-in method. In the reverse method, PPO-5CC was added to the solution of EDA and TEDA in methanol in portions during 30 min periods. Methanol was removed in the same manner as in the all-in method. The forward method was conducted contrary to the reverse method: PPO-5CC was first dissolved in methanol with TEDA, and EDA was added to the solution in portions during 30 min periods, then methanol was removed in the same manner as in previous methods. Samples were named NIPU-1, NIPU-2, NIPU-3, and NIPU-4, respectively, and their characteristics are given in Table 1 [25].

**Figure 3:**
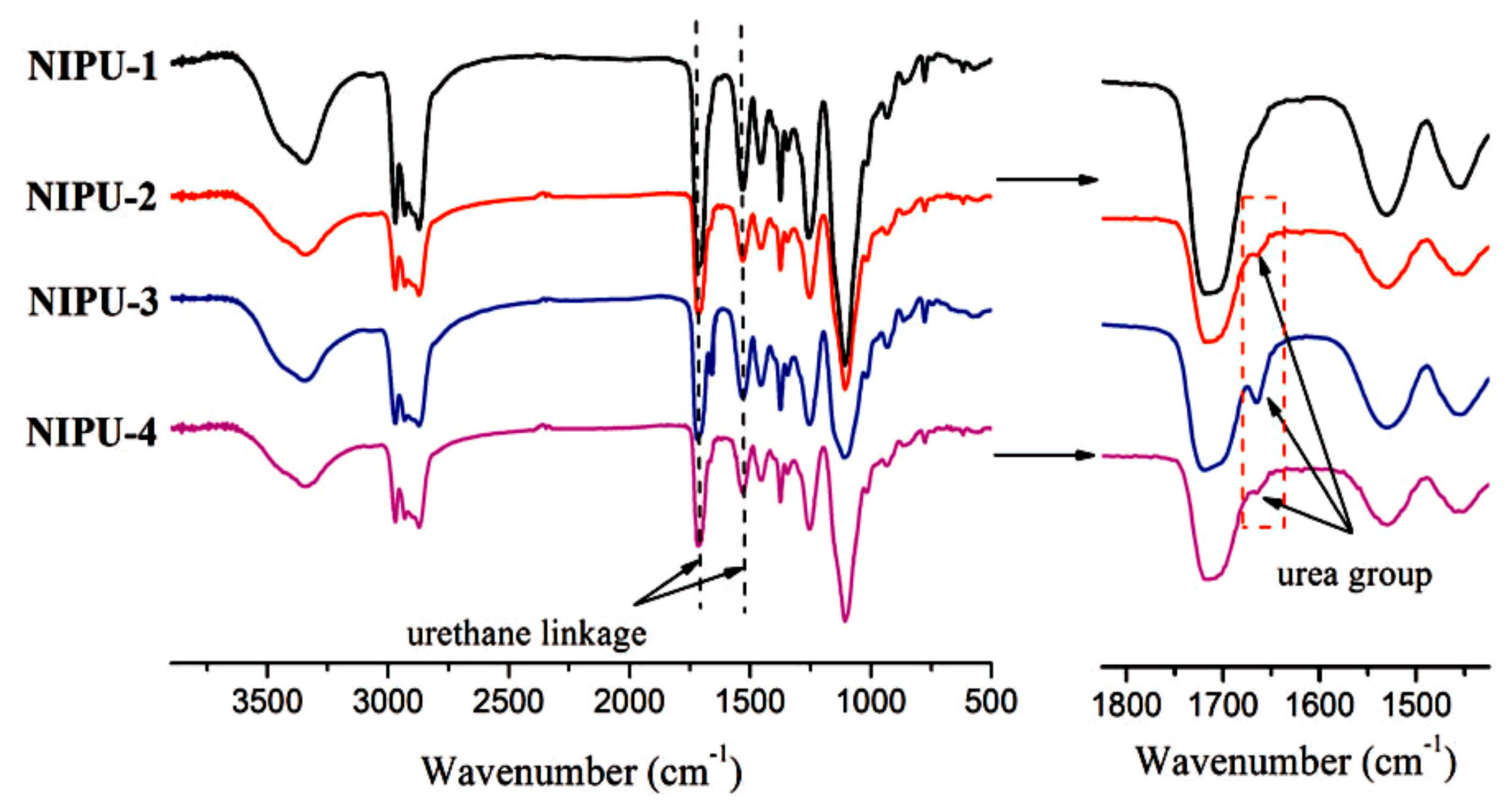
The FTIR spectra of NIPU prepolymers [25].

Based on Fourier transform infrared spectroscopy (FTIR) analysis, the influence of methanol on the structure of NIPU pre-polymers was proposed, as well, the role of the introduction method of substrates was discussed—Figure 3 [25].

Different ways of reactant introduction were utilized during the preparation process to reduce the urea content, leading to the formation of various NIPU structures. Altering the introduction mode of reactants improves the molar mass of the NIPU pre-polymers (Table 1) and reduces the urea content. Methanol was chosen as a solvent due to its effect on hydrogen bonding formation between NIPU chains. NIPUs under investigation achieved higher molar masses when obtained by the same type of method under the influence of methanol (NIPU-1 and NIPU-2). The presence of methanol enhances chain mobility and, at the same time, hinders hydrogen bonding between polyurethane chains. Over sufficiency of amine, molecules attack the urethane linkage, which leads to the formation of urea (band at 1665 cm^−1^). It is believed that the hydrogen bonding enhances the stability and provides shielding for prepolymer in the presence of surplus amine, as methanol causes said hydrogen bonding to be weakened. Due to that phenomenon, the peak of urea at 1665 cm^−1^ was the weakest for NIPU-4, compared to NIPU-2, while it was the strongest for NIPU-3. Furthermore, NIPU-3 showed the highest polymer dispersity index due to the higher urea content than in other NIPU compositions. Bisphenol-A diglycidyl ether (BADGE) was later on used as the cross-linking agent to obtain HNIPU materials. A significant upgrade in tensile strength and dynamic results were noticed for the HNIPU-4 (derived from NIPU-4), with an outstanding tensile strength of 10.8 MPa and elongation at break of 167% [25].

### 2.2. NIPU Synthesis Using other Approaches

As mentioned before, in addition to polyaddition between cyclic carbonates and amines, there are some other routes of obtaining non-isocyanate polyurethane materials. They are based on various types of reactions, namely, polycondensation, ring-opening polymerization, and rearrangement [1,13]. In the following paragraph, alternative ways of polyaddition will be discussed.

#### 2.2.1. Transurethanization Pathway (Polycondensation)

**Scheme 9:**
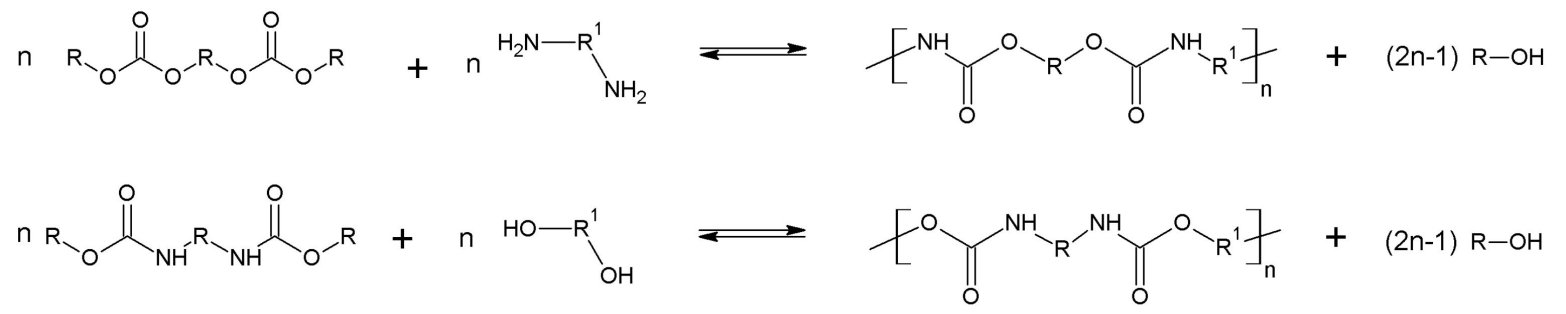
Route of obtaining NIPU by polycondensation of polycarbamate and diol or polycarbonate and diamine.

A transurethanization reaction involving polycarbamate and polyol yielding non-isocyanate polyurethanes (Scheme 9) is less environmentally-safe, as polycarbamates are mainly produced from phosgene. An alternative polycondensation route is conducted via reaction between at least difunctional carbonate and diamine (Scheme 9) [12].

**Scheme 10:**
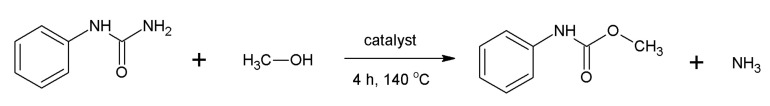
Synthesis of methyl N-phenyl carbamate using phenylurea and methanol [26].

Some sustainable approaches to produce polycarbamates involve nitro compounds, oxidative amines, or urea. The reaction of ureas with alcohols using various catalytic systems has been performed. For instance, Wang et al., have synthesized methyl N-phenyl carbamate using phenylurea and methanol—Scheme 10. PbO as a catalyst was found to be the most effective among various catalysts tested [26].

**Scheme 11:**
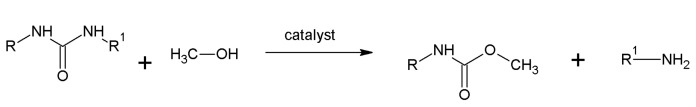
Synthesis of methyl carbamates from urea and methanol [26].

Yang and co-workers also studied polycondensation utilizing ureas. They obtained methyl carbamates from urea and methanol using a variety of catalysts—Scheme 11 [27].

**Scheme 12:**
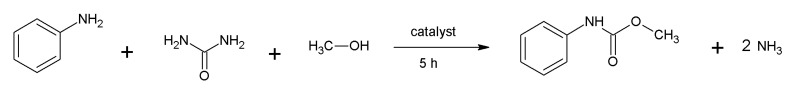
Synthesis of phenyl methyl carbamates utilizing aniline, urea, and methanol [26].

Qin et al., conducted a synthesis of phenyl methyl carbamates through the reaction of aniline, urea, and methanol—Scheme 12. Among various kinds of catalysts tested, they found that zeolite HY modified with KNO_3_ gave the best yields [28].

Non-isocyanate-based polyurethanes were also obtained by the polycondensation of amines, halides, and carbon dioxide, utilizing Cs_2_CO_3_ and tetrabutylammonium bromide. Various diamines and dihalides were examined, and the obtained NIPUs possessed all the properties synonymous with that of the conventional polyurethanes. However, utilizing polycondensation on a commercial scale is limited due to several reasons: the requirement of a catalyst, prolonged reaction time, necessity of purification of final polymers, and formation via side-reactions low *M_w_* compounds that may be toxic [23,29,30].

#### 2.2.2. Ring-Opening Polymerization

Ring-opening of 6–7 membered cyclic carbamates also leads to the formation of NIPUs. The product yield depends on the temperature of the reaction, preferably around 200 °C, and sodium hydride or N-acetyl caprolactam are used as catalysts. As the temperature grows, the CO_2_ content in the copolymer also surges, alongside the viscosity. Extensive studies are performed to devise cyclic carbamate synthesis in accordance with green chemistry postulates. Unfortunately, the current method involves phosgene, and so the overall process is not less harmful than the conventional method of obtaining polyurethanes [30].

#### 2.2.3. Rearrangement

Various rearrangement reactions may be utilized to obtain NIPUs. They include Curtius, Hofmann, or Lossen rearrangements. Unfortunately, the isocyanate is received in the course of the process, and so these methods are still not less toxic than the conventional approach. Moreover, halogens (especially bromine and chlorine) increase the amount of toxic waste produced. Recently, a toxic-free method for obtaining polyurethanes by utilizing the Lossen rearrangement was proposed using dimethyl carbonate as an activation compound in the environment of methanol and a tertiary amine in the role of the catalyst [30,31,32].

The synthesis of NIPUs is possible via various routes. Although there are several interesting methods for obtaining non-isocyanate polyurethanes (e.g., polycondensation, ring-opening, rearrangement, etc.), only the reaction of cyclic carbonates with amines has the potential to be introduced on an industrial scale, as it does not have as many obstacles as other abovementioned methods. Potential of this method is also reflected in the number of publications when compared to other routes. Compounds bearing cyclic carbonate moieties may be obtained from a wide range of precursors, which enables tailoring of the properties of final products. The possibility of using bio-based resources in the preparation of cyclic carbonates is an additional advantage in favor for taking this synthetic route. Also, conversion of oxirane rings into five-membered carbonate moieties utilizes carbon dioxide, which, at higher concentrations, acts in the atmosphere as a pollutant. NIPUs structure can be tailored by use of aliphatic or aromatic types of amines, characterized by different reactivities. The choice of other chemicals used in the process of obtaining NIPUs, such as solvents or catalysts, is also of great importance and has been a subject of separate studies. Overall, there is still plenty of room for further investigations on the topic of non-isocyanate polyurethane synthesis.

## 3. NIPU Composites

As conventional PUs are broadly modified using different types of reactive and non-reactive additives, this approach can also improve NIPU properties. NIPUs bearing primary and secondary hydroxyl side groups can undergo chemical modification, including grafting reactions and cross-linking with chemical agents able to react with OH- moiety.

In most cases, the synthesis process and the properties of obtained NIPU and NIPU composites are evaluated in the same way as conventional PU or any other polymeric materials. Characterization of substrates or products and monitoring of the process of synthesis are generally done by utilizing Fourier transform infrared spectroscopy (FTIR), nuclear magnetic resonance spectroscopy (NMR), X-Ray diffraction spectroscopy (XRD), scanning electron microscopy (SEM), and size exclusion chromatography (SEC). The methods used for mechanical property appraisal are tensile strength tests (measuring tensile strength itself, Young’s modulus, and elongation at break), impact strength tests, and various hardness tests (pencil hardness or Shore hardness). Thermal properties are usually tested using thermogravimetric analysis (TGA) and differential scanning calorimetry (DSC). Dynamic mechanical analysis (DMA) is performed regularly to determine thermo-mechanical properties. Chemical property tests include, but are not limited to, resistance for acid, alkali, various solvents, hydrolytic stability, water uptake, and anti-corrosive properties.

### 3.1. NIPU as a Polymer Matrix for Nanocomposites

Research interest has been focused recently on NIPU nanocomposites. They show improved properties with an addition of the nanofiller in the small amounts of 1–5%, which is much less than in the case of traditional micro-structured composites that often require much higher filler loads.

**Scheme 13:**
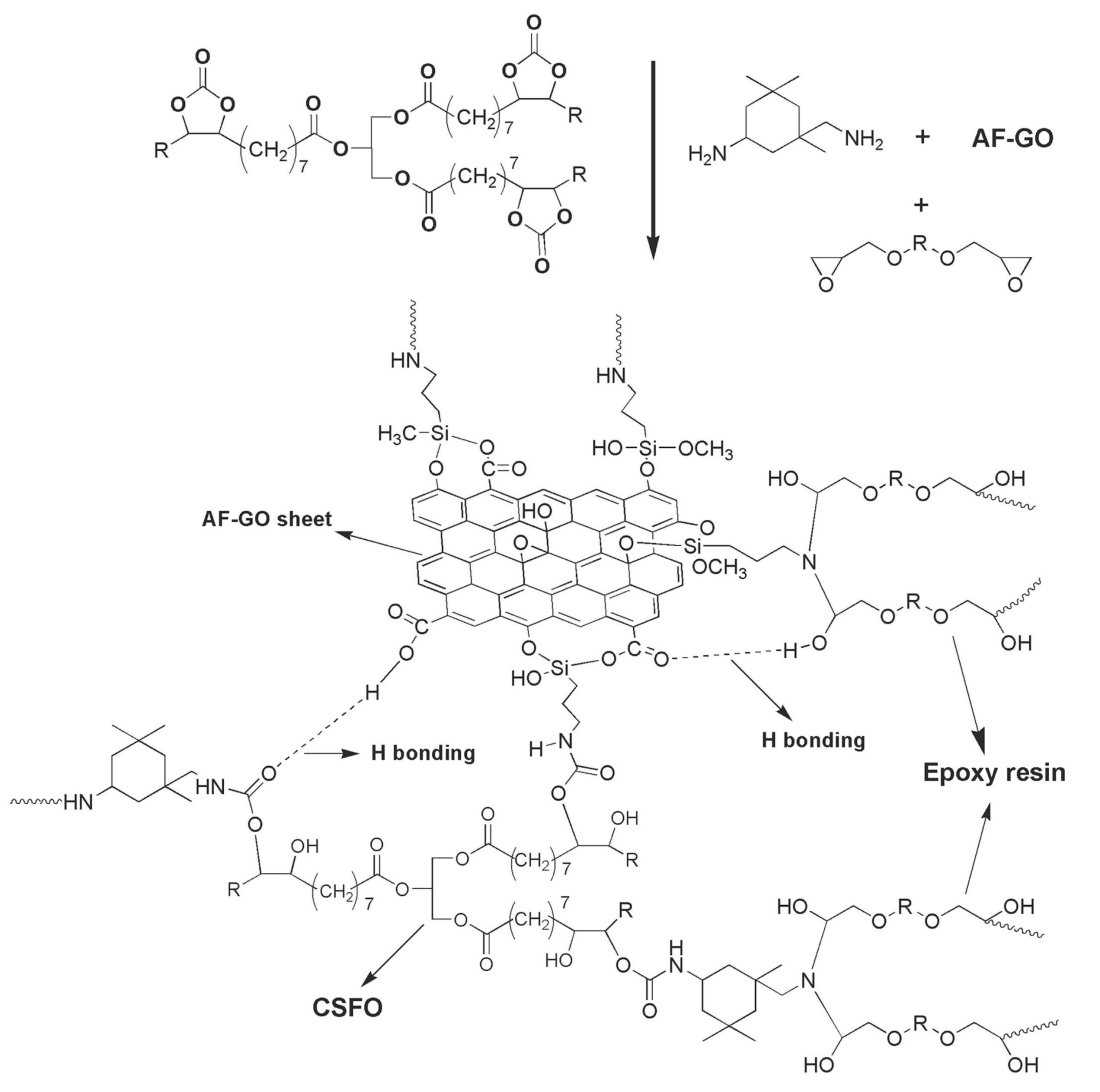
Plausible interactions of AF-GO with the polymer matrix [33].

Along this line of interest, Dolui et al., devised a set of (NIPU)-blend-epoxy hybrid materials (HNIPU) based on sunflower oil that was reinforced by amine-functionalized graphene oxide (AF-GO). Usage of sunflower oil, a bio-basted raw material, benefits an already environmentally-friendly concept of non-isocyanate polyurethanes. Carbonate compound for HNIPU was obtained in the process of combining epoxidized sunflower oil (ESFO) and carbon dioxide (CO_2_) in pressurized conditions 50 bar and the temperature of 120 °C. Carbonated sunflower oil (CSFO) bearing cyclo-carbonate moieties was obtained and later mixed with commercially available epoxy resin (up to 30 wt% in relation to CSFO). Isophorone diamine was used as a curing agent in solvent reaction at 70 °C in THF, followed by curing and post-curing at 90 °C and 130 °C for 48 h and 1 h, respectively. The HNIPU with 30 wt% epoxy was chosen to be modified with amine-functionalized graphene oxide, as it showed the best mechanical properties. The overall route of the synthesis, modification, and plausible interactions between polymer matrix and nanofiller are shown in Scheme 13 [33].

All the obtained nanocomposites (0.3, 0.6, and 1.0 wt% of AF-GO) showed improvement in mechanical properties in comparison to the unmodified polymer. Mechanical and other properties, such as thermal stability and flame retardancy, have improved with the addition of AF-GO. Superior properties of the composite materials with nanofiller may be attributed to hydrogen bonding and covalent interconnection of amine-modified graphene oxide with the polymer matrix. Enhancement of the properties of nanocomposites increases with AF-GO content. The same research group developed other types of NIPU composites using amine-functionalized multi-walled carbon nanotubes (AF-CNT) in the place of graphene oxide. As previously stated, carbonated sunflower oil (CSFO) and isophorone diamine (IPDA) were used [33].

**Scheme 14:**
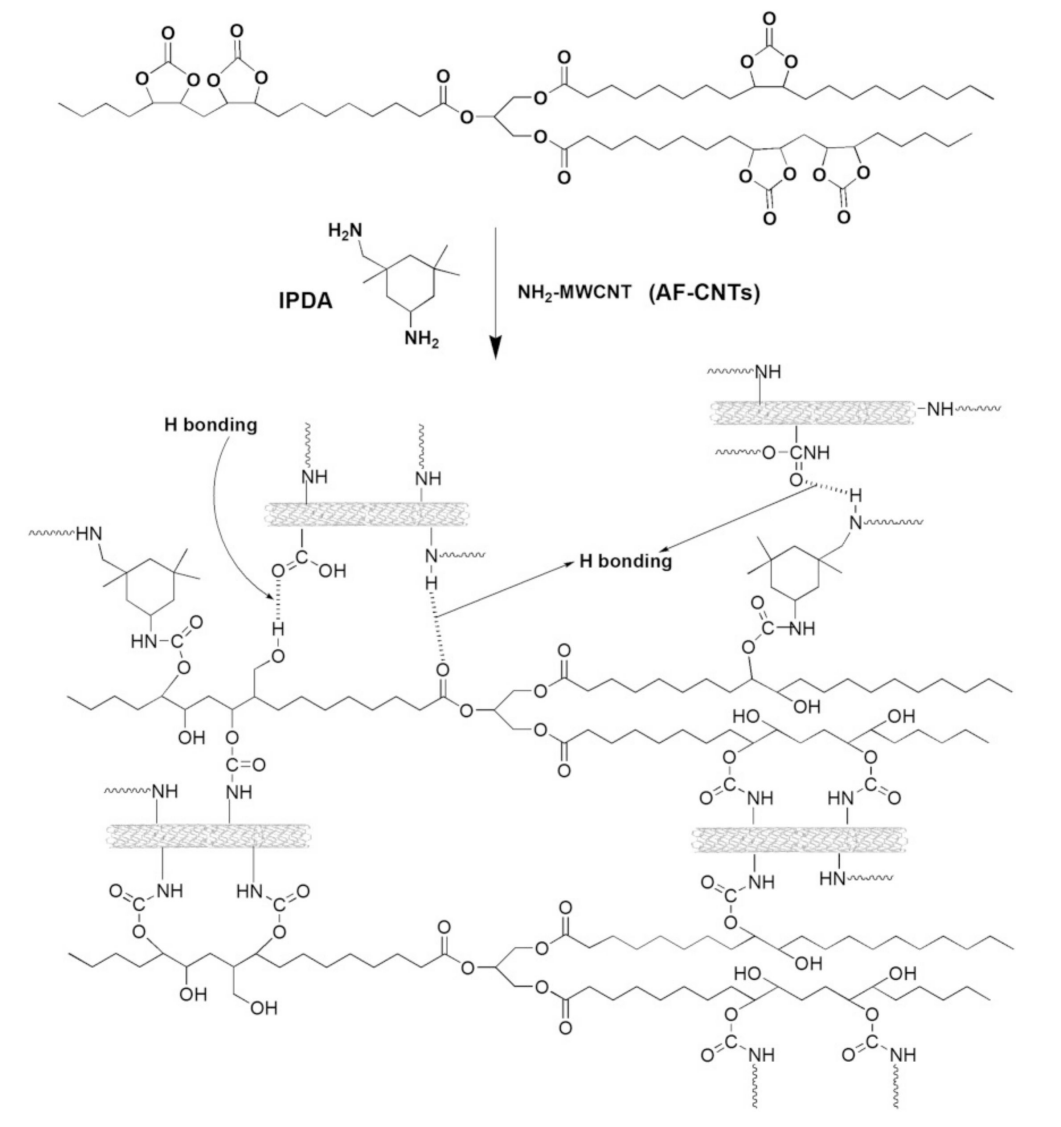
Plausible cross-linked structure of the CSFO/IPDA/AF-CNTs nanocomposites [34].

In the preparation protocol, multi-walled carbon nanotubes were first treated with concentrated nitric acid and ultra-sonicated. After additional processing (filtration, purification, and drying), functionalized carbon nanotubes (f-CNTs) were aminated by the addition of 3-amino propyl trimethoxy silane (APTMS). The obtained amine-functionalized multi-walled carbon nanotubes (AF-CNT) were then used as reactive filler in wt% of: 0.50, 1, 1.5, and 2 in relation to the weight of CSFO—Scheme 14 [34].

Properties of the obtained nanocomposites improved compared to reference NIPUs, which is explained by the presence of strong hydrogen interactions and covalent interlinkage of AF-CNTs with CSFO/IPDA polymer networks. The performance of obtained materials was enhanced with the loading of AF-CNTs up to 1.5 wt%. The thermal and mechanical features improved significantly, and the limiting oxygen index reached 30%, which suggests the synthesized compositions show self-extinguishing traits. The initial decomposition temperature was determined to be 274 °C, while the temperature of 50% weight loss was measured to be 390 °C for composition without any addition of CNTs. It reached 310 °C for the initial decomposition step, as well as 430 °C for 50% weight loss for composition with 1.5 wt% of CNTs [34]. The value for non-filled material is higher than conventional PU materials based on sunflower oil (initial decomposition temperature ca. 250 °C, 50% weight loss at ca. 380 °C) [35].

As physical-chemical properties improved, the electromagnetic interference shielding effects of the sample with 1.5 wt% AF-CNTs were tested. The assessed sample exhibited microwave absorption of 217 dB at 10.86 GHz in the 8.2–12.4 GHz (X-band) frequency range while being 3 mm thick, suggesting NIPU/CNT nanocomposites show promise for screening utilization and shielding against electromagnetic radiation [34].

Liu et al., obtained a batch of non-isocyanate polyurethane films in the process of reacting gallic-acid-based cyclic carbonate with difunctional amines. Further modification with polyhedral oligomeric silsesquioxane (POSS) particles bearing oxirane moieties yielded NIPU/POSS materials, with POSS covalently incorporated in the polymeric matrix. The gallic-acid-derived hybrid samples exhibited extraordinary mechanical and thermal properties (impact strength, adhesion, flexibility, pencil hardness), yet water resistance was not satisfying. The incorporation of POSS into polyurethane matrices improved pencil hardness, water resistance, and thermal performance of the obtained hybrid films, although their adhesion decreased slightly. As the thermal decomposition of unmodified NIPUs occurred at similar temperatures (around 360 °C) as for conventional PU, compositions with POSS particles were more thermally stable (decomposition temperature of ca. 380 °C) [35,36].

Kathalewar et al., prepared NIPU composites with an addition of surface-treated ZnO particles. The modification of ZnO was conducted via an aqueous precipitation method with cyclic carbonate functional alkoxy silane. This approach allowed surface-treated ZnO (TZnO) particles to bear pendant cyclic carbonate moieties and, in turn, function as a cross -linking agent for the matrix. NIPU matrix was obtained using cyclic carbonate derivative of epoxy resin (C-GY) and 4,9 dioxadodecane-1,12-diamine as a hardener, and dimethyl formamide was used as a solvent. Treated and untreated ZnO particles were added to the matrix in a *w*/*w* ratio of 1%, 2%, and 3%. The formulations were dissolved in a mixture of xylene and dimethyl carbonate, applied on mild steel and aluminum plates, and underwent a curing process at 70 °C and 135 °C for a total of 1.5 h. A conventional polyurethane formulation (CPU) based on acrylic polyol and N-3390 cross-linking agent was also prepared to be compared to ZnO–NIPU composites and NIPU coating without the addition of any particles (B-NIPU) [37].

All obtained coatings were tested for mechanical and chemical properties. Overall properties of NIPU composited were expected to be influenced by the cross-linking of TZnO particles. Pencil hardness, cross hatch adhesion, and flexibility turned out to be similar in the case of all formulations. Interestingly, CPU flexibility was lower than NIPU coating without the addition of ZnO particles. It was attributed to the chemical structure of both formulations as CPU had a more cross-linked structure due to the usage of trifunctional (HDI-based) N-3390 cross-linking agent. The falling ball impact method was used to evaluate the impact resistance of obtained coatings. All NIPU coatings showed impact resistance of 70.8 lbs, while CPU achieved impact resistances of 53.1 lbs and 47.2 lbs in intrusion and extrusion testing methods, respectively. The scratch hardness of the CPU coating was similar to 3% TZnO coating. However, as the loading of the ZnO filler in NIPU compositions increased from 1% to 3%, their scratch hardness also improved. In the case of treated ZnO particles, it was attributed to higher cross-linking of the polymer network, as cyclic carbonate functional groups in the TZnO reacted with amine compounds. In contrast, untreated ZnO was present in the matrix only in the form of suspended particles [38,39]. This trend was also observed for abrasion resistance. Among obtained coatings, the highest abrasion resistance was observed for composites containing 3% TZnO NIPU formulation (3.3 mg weight loss). The abrasion resistance of untreated NIPU (5.1 mg weight loss) was superior to CPU (7.8 mg weight loss). The results of the dynamic mechanical analysis also corresponded with other testing methods. As the loading with ZnO nanofiller increased, the storage modulus of all compositions also improved. Yet again, surface-treated ZnO particles performed better than untreated ones. Glass transition temperature (*T_g_*) increased alongside the loading of treated and untreated ZnO particles, which was attributed to the restricted mobility of the matrix chains caused by interactions between the filler and NIPU [37,40].

Obtained coatings were also tested for their chemical resistance by various testing methods. All of the formulations showed superb resistance to both acid and alkali, evaluated by the immersion method. It was believed that hydrogen bonding had a shielding effect on hydroxyl groups present in the NIPU matrix, and thus, its acid resistance was not diminished. Excellent results were also obtained for solvent resistance in MEK and xylene. The hydrolytic stability was tested by submersion of obtained samples in boiling water for 8 h. Due to the presence of secondary hydroxyl groups in nano composites with ZnO and TZnO, their hydrolytic stability was superior to the conventional polyurethane. Moreover, loading of the NIPU matrix with ZnO fillers did not cause any unwanted porosity. The salt spray method was used to evaluate corrosion resistance. NIPU composite coatings did not show any damage after 500 h of salt spray test at 35 °C, while B-NIPU showed only minor corrosion of metal surface under the cross mark made on the coating. The results were comparable to those of conventional polyurethane [37].

Fleischer et al., prepared composites filled with cellulose carbonate and matrices obtained from carbonate compound derivatives of glycerol (GGC), trimethylolpropane (TMC) and pentaerythritol (PEC) glycidyl ethers, and citric acid amino amides (CAA), based on triethyl citrate hexamethylene diamine (HMDA). The fact that most of the listed materials are bio-based only benefits the idea of NIPUs being environmentally friendly materials. Respective cyclic carbonates were mixed with HMDA or CAA and, subsequently, cured at 70 °C for 8 h. For the preparation of composites, cellulose filler was mixed with carbonate and amine compounds and cured under the same conditions. Such a synthetic route is a convenient method to obtain non-isocyanate polyurethanes and their composites according to green chemistry regulation. Thermal and mechanical properties were evaluated for obtained NIPU and their respective composites with cellulose. Glass transition temperatures were 20 °C for GGC–HMDA, 47 °C for TMC–HMDA, and 51 °C for PEC–HMDA. When NIPUs were obtained with citric acid amino amides, their *T_g_* increased by 16 °C for GGC–CAA and 9 °C for TMC–CAA. *T_g_* of PEC–CAA remained unchanged. Young’s modulus of both GGC-based NIPUs was uniform at 7 MPa. On the contrary, it increased from 590 MPa for TMC–HMDA to 1230 MPa for TMC–CAA and 660 MPa for PEC–HMDA to 1740 MPa for PEC–CAA. There is an observable trend for all of the mentioned values: the usage of citric acid amino amides as curing agents rather than hexamethylene diamine results in increasing in glass transition temperature and Young’s modulus. Tensile strength shows the same trend while elongation at break lowers. All those interactions are believed to be due to the tri-functionality of CAA and di-functionality of HMDA. In terms of carbonate compound structures, both GGC-based (difunctional)NIPUs are flexible, as their structure is much less cross-linked than TMC– and PEC–NIPUs (both are tri-functional). However, when GGC is blended with other carbonates, interesting synergies are observed. PEC–GGC 3:1 *w*/*w* mixture of carbonates reacted with HMDA gives NIPUs with tripled stiffness and tensile strength compared to the values of the single carbonate compositions. For blends cured with CAA, the results are alike. An extensive explanation of the genesis of this blend synergism may be done as more complex research is to be conducted. The carbonate blend of TMPC–GGC 1:2 *w*/*w* was chosen to be modified with cellulose and cellulose carbonate. In both formulations, the polymer matrix was loaded with 5 wt% of filler. Cellulose carbonate–NIPU composite showed Young’s modulus of 2600 MPa in comparison to 2100 MPa for non-filled NIPU. Tensile strength and elongation at break were almost unchanged. Better compatibility of carbonated cellulose with NIPU matrix is reflected in the improvement of Young’s compared to unmodified cellulose filler, as cellulose carbonate can react with NIPU matrix via urethane linkages. Other carbohydrates, e.g., nanocellulose can also be added to NIPU matrices via this method [41].

**Scheme 15:**
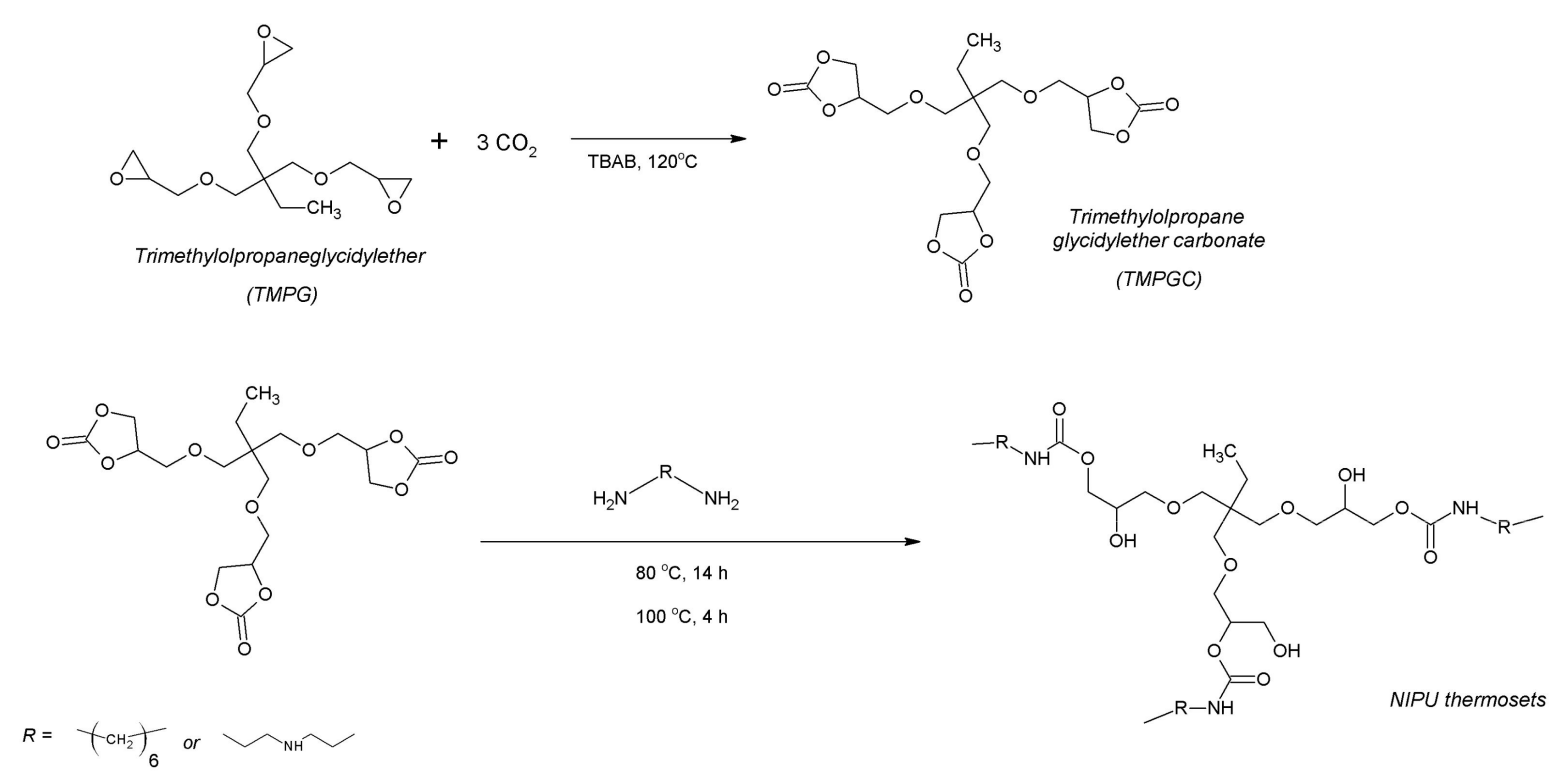
Formation of NIPU thermosets from TMPGC, HMDA, or DETA [42].

Yang et al., prepared and studied NIPU composites filled with corundum (Al_2_O_3_) and silicon carbide (SiC). Ceramic fillers were also covered with various carbon sources (namely dopamine, glucose, and graphene oxide) and, subsequently, thermally treated to produce hybrid materials bearing a graphene outer layer with ceramic material inside. NIPU matrix was obtained from trimethylolpropane glycidyl ether carbonate (TMPGC) and hexamethylene diamine (HMDA) or diethylenetriamine (DETA) as curing agents. NIPU composites were prepared by mixing graphenatedated ceramic fillers with carbonate substrate and later addition of either HMDA or DETA. The curing process was conducted for 14 h at 80 °C, and later on, the post-curing process was applied for 4 h at 100 °C (Scheme 15) [42].

Composites with 10 or 30 wt% of selected fillers were evaluated for their thermal and mechanical properties. The reference DETA-cured NIPU had Young’s modulus of 3900 MPa. A Young’s modulus of 7800 MPa was achieved for its composite filled with 30 wt% of corundum filler untreated with graphene. However, tensile strength and elongation at break were observed to be lowered (from circa 100 MPa to 90 MPa and from 3% to 1.2%, respectively) due to the stiffening effect of the filler. NIPU-bearing hydroxyl and amine groups were believed to interact with Al cations present in corundum. When filler particles were graphenated, reduced interfacial adhesion was thought to be the cause of worsening stiffness and tensile strength of obtained materials. In the case of SiC filler, significant enhancement of Young’s modulus (to 5100 MPa from 2400 MPa) was observed for graphenated-particle-based dopamine, with TMPGC–HMDA matrix at 10 wt% content of the filler. In terms of thermal properties, utilizing differential scanning calorimetry, differences in *T_g_* between reference NIPUs and their composite materials were not significantly high (up to ±5 °C). Two-point electrical conductivity measurements were undertaken to assess the electrical properties of obtained composites. Only samples obtained with diethylenetriamine showed electrical conductivity when filled with 30 wt% graphenated fillers. Thus, such low electrical conductivity may be the base for obtained materials to be eligible for electromagnetic shielding applications [42].

Pössel et al., prepared NIPU composites containing γ-Al(OH)_3_ (O-gibbsite) nanoplatelets, which were subsequently modified by surface treatment with lysine to obtain ly-gibbsite nanofiller. Utilizing the freeze-drying method, a gibbsite was obtained in the form of a powder. To facilitate better compatibility with the non-isocyanate matrix, gibbsite was treated with L-lysine, which was done before the freeze-drying process. Two carbonate compounds: trimethylolpropane glycidylethercarbonate (TMPGC) and pentaerythritol glycidylethercarbonate (PGC), as well as two amine compounds: hexamethylenediamine (HMDA) and diethylene triamine (DETA), were used for the synthesis of the matrices. Due to the volatile nature of amine compounds, the first step of composite preparation was to disperse gibbsite fillers in the cyclic carbonate substrate by a high shear mixer. After the addition of respective amine, NIPU materials were cured at 80 °C overnight and underwent a post-curing process for 4 h at 100 °C [43].

Thermal and mechanical attributes were determined for obtained samples. Effects were observed to be similar to other NIPU composites [42]: filling the polymer matrix with O-gibbsite (untreated with L-lysine) caused Young’s modulus to increase by 180% for TMPGC–HMDA, 140% for TMPGC–DETA, and 150% for PGC–DETA, respective to their non-filled counterparts. At the same time, tensile strength and elongation at break were observed to diminish. Modification with lysine proved to improve the performance of gibbsite fillers. For example, loading NIPU composites with up to 40 wt% of ly-gibbsite caused Young’s modulus to improve from 2800 MPa for TMPGC–HMDA to 6000 MPa for its composite. Improvement of similar magnitude was also observed for TMPGC–DETA- and PGC–DETA-based materials. Thin coatings (100 μm) were prepared with obtained NIPU composite formulations. Samples with untreated O-gibbsite happened to be opaque due to the formation of large O-gibbsite agglomerates. On the contrary, ly-gibbsite–NIPU composite remained translucent, even when the content of filler was 40 wt%. Ly-gibbsite was also used as a means to improve flame retardancy for NIPU. While halogen-containing compounds are known to be used for such applications, avoiding them comes in with accordance to green chemistry postulates. Using a gas burner to set NIPU composite samples aflame was conducted as a means to assess their properties. A flammable cotton piece was set below the burning samples to check for fire spread possibilities. Without any surprise, reference NIPUs turned out to be highly flammable, with burning droplets dripping down on the cotton and setting it aflame. Ly-gibbsite added in the amount of 30 wt% to TMPGC–HDMA and PGC–DETA matrices significantly enhanced their flame retardancy [43].

### 3.2. NIPU as an Additive

Apart from being polymeric matrices, NIPUs may act as additives, modifiers, or fillers incorporated into different polymers. In such a way, new material properties can be achieved by utilizing NIPUs’ inherent properties and the formation of favorable interactions with the polymer matrix.

For instance, non-isocyanate polyurethane (NIPU) was applied to solve the brittleness issue of poly(propylene carbonate) (PPC), which shows poor elongation at break (below 10% at 20 °C) that critically restricts its applications. Thus, separate NIPUs were obtained and introduced to PCC to enhance its properties. NIPU was chosen due to its non-toxic synthesis, low glass transition temperature, and capability to create hydrogen bonding between carbonyl or oxygen units in poly(propylene carbonate). After the synthesis, NIPU and PCC were melt-blended in a mixer in a variety of different weight ratios (NIPU/PPC = 1/99, 2.5/97.5, 5/95, 8/92,10/90, 13/87, or 15/85). The abovementioned ratios of NIPU/PCC had a significant impact on the miscibility and the morphology of obtained materials, which was connected with the equilibrium between inter and intermolecular hydrogen linkages formed between poly(propylene carbonate) and polyurethane. As the amount of NIPU in the composition grew, the fraction of intermolecular hydrogen linkages between two copolymers also increased. Below the amount of 10 wt%, NIPU dispersed equally in poly(propylene carbonate), and the shift from brittle to durable occurred when L/d reached a critical value of 1.74, where L and d were center-to-center distance and the diameter of the particles, respectively. The unnotched impact strength grew three times (to above 20 KJ/m^2^) compared with neat PPC (slightly above 8 20 KJ/m^2^) when the amount of introduced NIPU reached 10 wt%. Flocculation of NIPU was observed when the NIPU loading reached 13 wt%, which lead to a decline in toughness. Elongation at break for PCC was measured to be around 10%, but while modified with NIPU, it increased up to 30% when filler loading was over 8 wt%. It increased tensile strength (from around 53 MPa for PPC) by 15 MPa for formulations filled with 2.5 wt% of NIPU. On the contrary, elongation at break and the tensile strength diminished with the addition of 13 wt% of NIPU. Such a phenomenon was noticed to occur in PLA/hyperbranched polyamide blend [44] and PPC/low-molecular-weight urethane [45].

Mechanical properties, chemical structure, and morphology of an epoxy resin modified by combining nanoclay nanoparticles NIPU were studied by Białkowska and co-workers. The use of nanoclays as strengthening fillers for epoxy resins has already been widely studied. However, not much research has been done to reinforce epoxy resins by incorporating non-isocyanate polyurethanes. Based on preliminary studies, epoxy-nanoclay nanocomposite containing 1 wt% of bentonite was chosen for further modification with different amounts of NIPU, yielding hybrid epoxy composites. As for the NIPU, an MNF 30 oligomer (i.e., oligomer based on urea and formaldehyde, containing 30% mol of 2-hydroxy-6-naphthalenesulfonic acid [HNSA], with an equimolar proportion of hard and soft segments) was used due to its outstanding tensile strength (reaching 12.7 MPa) and a sufficient elongation at break, reaching 110%, amongst compositions tested before [46].

**Figure 4:**
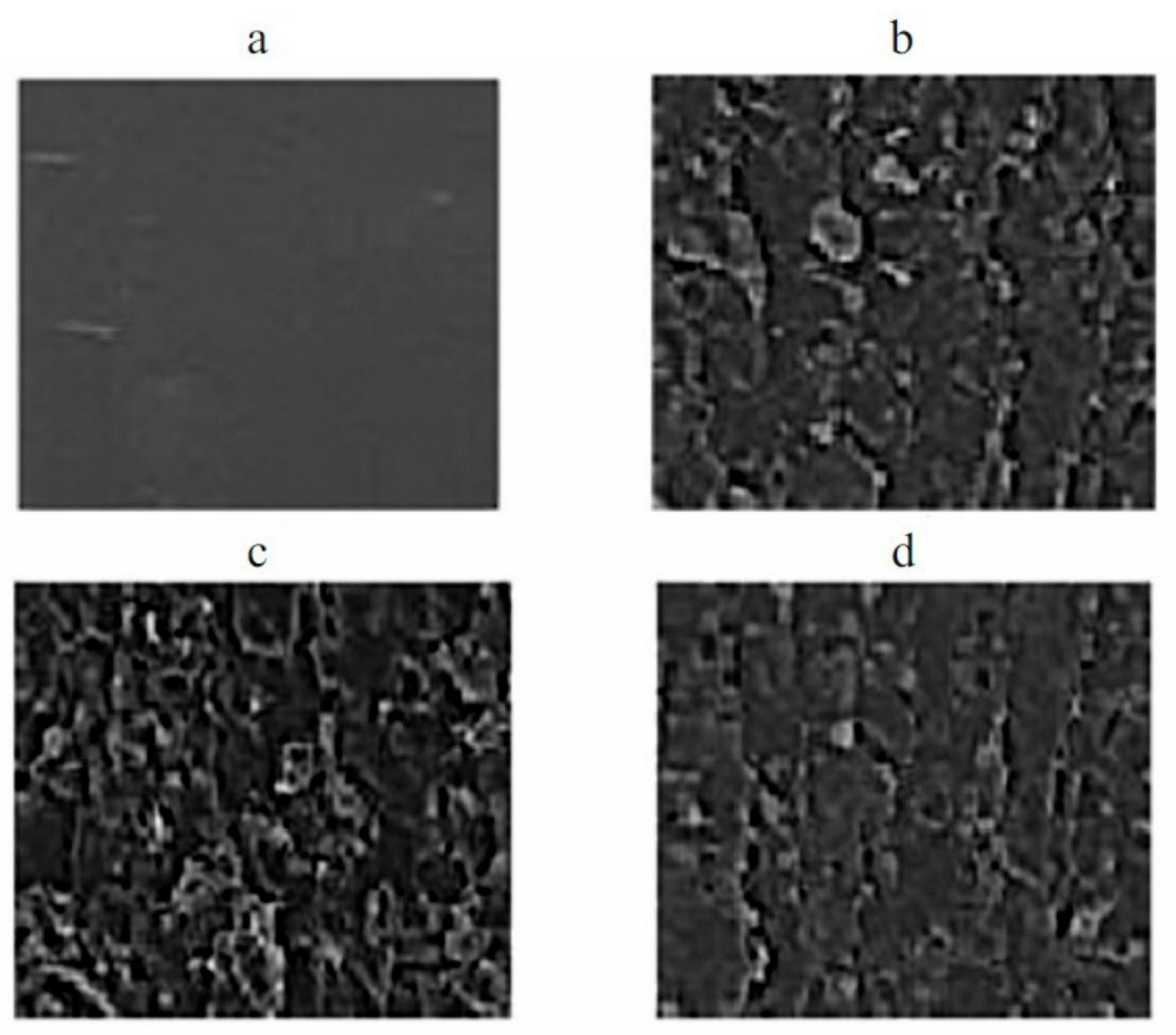
SEM micrographs (magnification 1500×) of fracture surfaces of samples containing the unmodified epoxy resin (**a**), 5% NIPU and 1% Nanobent (**b**), 10% NIPU and 1% Nanobent (**c**), and 15% NIPU and 1% Nanobent (**d**) [47].

Nanoclay was mixed in, becoming a 15% dispersion in acetone. Ultrasonic homogenization was applied to ensure the uniformity of the mixture. Oligomeric urethane was added in up to 15 wt% in its liquid state. Finally, a stoichiometric amount of curing agent was added and mixed, and afterward, the compositions were put inside aluminum molds, ensuring shapes preferred for later mechanical testing. The curing process was conducted at room temperature, followed by post-curing at 60 °C. Tolerance to slow (the critical stress intensity factor *K_c_*) and fast crack propagations (the impact strength-IS) and the flexural strength in three-point bending were determined for samples with different amounts of NIPU. The epoxy formulations exhibited higher ductility, superior flexural strain at break, better flexural energy to break, together with brittle fracture energy. Compositions loaded with 10% NIPU and 1% clay exhibited the highest impact strength and *K_c_* among other samples (including reference). A negligible reduction in the flexural strength in comparison to the pure epoxy sample can be seen. However, a very significant increase in *ε_b_* is observed upon the incorporation of NIPU. The explanation for the improvement in the resistance to slow and fast crack propagations may be found by analyzing SEM micrographs (Figure 4). Hybrid epoxy composites displayed rugged and stratified fracture surfaces with notable plastic yielding, while the pure epoxy counterpart exhibited a smooth surface, ordinary for fragile polymers, without plastic yielding or an elongated structure [47].

### 3.3. NIPU as a Building Block in Copolymer Synthesis

Monomers such as esters, amides, and epoxy compounds are utilized to synthesize NIPU copolymer hybrid materials. Most attention is paid to epoxy compounds as they are often intermediates in obtaining cyclic carbonates from different kinds of raw materials, including those from renewable sources [6].

**Scheme 16:**
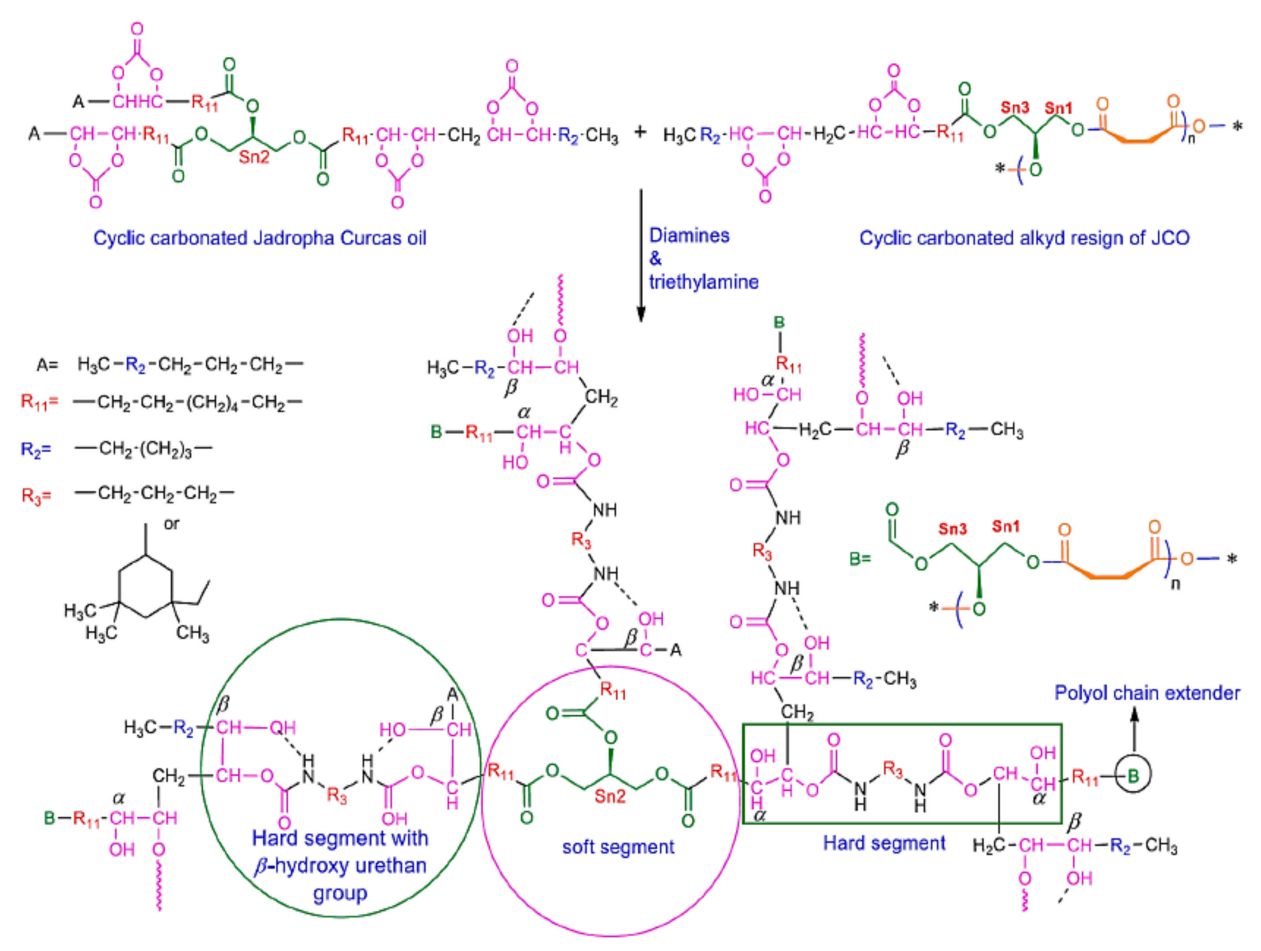
The cross-linked polymer network of obtained NIPU [48].

Hence, Haniffa et al., obtained non-isocyanate polyurethane by using 1,3-diaminopropane (DM) and isophorone diamine (IPDA) utilized in the role of curing agents. The raw material was Jatropha curcas oil (JCO). Substantial unsaturated fatty acid content and satisfactory oxidation stability of Jatropha curcas oil make it possible for this oil to be used for numerous NIPU commercial applications. Additionally, it is a renewable resource, which only adds to the concept of NIPUs being environmentally friendly materials. Carbonated JCO (designated CJCO) was synthesized through a two-step process. First, JCO was epoxidized with hydrogen peroxide, with formic acid being present in the reaction environment. Then, CJCO was subjected to carbon dioxide fixation with tetrabutylammonium bromide (TBAB) as a catalyst. Alkyd resin of JCO was also treated in the same way, leading to carbonated alkyd resin (CC-AR). CJCO was mixed with various ratios of CC-AR (*w*/*w*) to boost its characteristics and reacted with 1,3-diaminopropane (DM) and isophorone diamine (IPDA), as shown in Scheme 16 [48].

Thermomechanical properties of the obtained NIPUs turned out to be effectively altered alongside the CC-AR weight ratio and amine amount. The CJCO cured with DM displayed outstanding elongation at break of 230%, yet low Young’s modulus. CJCO/CC-AR mixed in 1:3 ratio showed circa threefold improved Young’s modulus of 680 MPa and better *T_g_* value of 44 °C with IPDA in relation to DM. The reason for this may be that the cycloaliphatic structure of IPDA creates steric hindrance, or it is due to a highly cross-linked network structure. However, CJCO/CC-AR blend with CC-AR ratio of 1:4 exhibited a decrease in modulus and tensile strength in relation to NIPU-based composition with a lower CC-AR ratio. Thus, the further increase of CC-AR in the CJCO/CC-AR blend is believed to result in slipping amongst the chains of the saturated matrices alongside the further reduction in terms of thermomechanical properties [48].

**Scheme 17:**
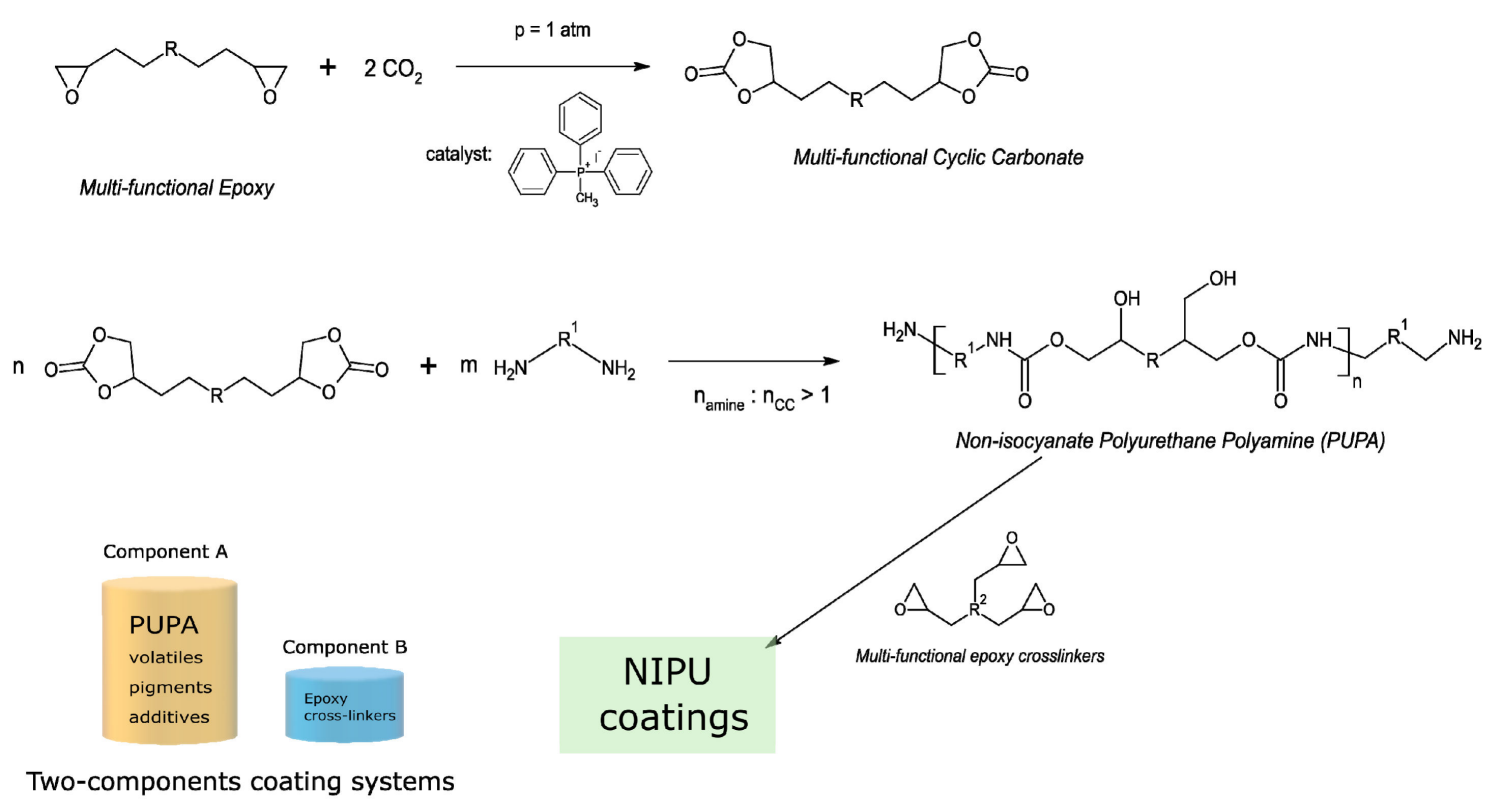
Development of NIPU coatings [49].

Asemani et al., obtained NIPU coatings by (1) synthesizing cyclic carbonates via catalytic fixation of CO_2_ by epoxy compounds and then (2) by reacting them with an excess quantity of amine to, in turn, obtain polyurethane polyamines (PUPAs). Final products were prepared by mixing stoichiometric amounts of PUPAs and aliphatic epoxies as cross-linking compounds—Scheme 17 [49].

The obtained results revealed that well-designed NIPU oligomers led to materials showing extraordinary performance, being flexible in low temperatures and resistant to chemical agents. Such customization is possible by choosing proper cyclic carbonate and amine types and varying their relative molar ratios. Generally speaking, non-isocyanate polyurethane coatings with mechanical, physical, and chemical properties similar to traditional polyurethane coatings can be obtained by deliberate choice of NIPU building blocks. On the contrary, the thermal stability of NIPU coatings was reported to be remarkably lowered in relation to traditional polyurethane coatings [49].

Pathak et al., synthesized a non-isocyanate polyurethane coating based on dehydrated castor oil fatty acid (DCOFA) and tris-2-hydroxyethyl isocyanurate (THEIC). Non-toxic THEIC provided excellent thermal and chemical resistance to the polymer due to its heterocyclic nature, enabling its usage in numerous practices in the manufacturing of coatings. The synthesis consisted of esterification of THEIC with DCOFA and subsequent epoxidation of the ester formed (TEFA), followed by fixating CO_2_ with oxirane rings within the intermediate product—THEIC–ester of fatty acid (CTEFA). Cyclo-carbonated THEIC–ester of fatty acid (CTEFA) underwent the curing process with either hexamethylene diamine (HMDA), isophorone diamine (IPDA), or diamino diphenyl sulphone (DDS). The type of amine used for curing had a significant influence on the overall properties of obtained coatings. Coatings with aromatic and cyclo-aliphatic amines used as curing agents exhibited superior mechanical, chemical, thermal, and anti-corrosive properties compared to those obtained with the aliphatic amine [50].

De Aguiar et al., synthesized polydimethylsiloxane hybrid urethane (PDMSUr) coatings by ring-opening polymerization of a PDMS-derived cyclic carbonate (CCPDMS) by 3-aminopropyltriethoxysilane (APTES) alone or with 5-amino-1,3,3-trimethylciclohexano methylamine (IPDA). Poly(dimethylsiloxane) diglycidyl terminated ether (E-PDMS) underwent a carbon dioxide fixation reaction with tetraethylammonium bromide (TEAB) as a catalyst, resulting in the formation of PDMS-derived cyclic carbonate (CCPDMS). The first synthetic route was a two-step reaction—in the first step, CCPDMS and 3-aminopropyltriethoxysilane (APTES) were stirred and heated at 70 °C for 40 min. Next, phosphotungstic acid PWA, added in the amount of 35 wt% or 1 wt%, dissolved beforehand in dimethylacetamide (DMAc), was mixed in. The reactants were stirred at 50 °C for 24 h. Another batch of the hybrid PDMSUr was obtained in three steps. First, CCPDMS and 5-amino-1,3,3-trimethylciclohexano methylamine (IPDA) were mixed at 70 °C for 20 min. Afterward, APTES was mixed in and stirred again for 20 more minutes. Finally, PWA (prepared in the same amount and manner as previously) was added, and the mixture was stirred 50 °C for 24 h, as it was done in the first route. Although authors primarily focused on the physical surface modification of biomedical grade metallic surfaces by oxygen plasma and laser treatment, the hybrid PDMS–urethane coatings were found to be fitting materials for covering metal surfaces utilized in orthopedic or dental implants, characterized by preferable adhesion strength. Additionally, the obtained hybrid-PU layers may serve as anti-corrosion materials for metals directly exposed to physiological media by forming a hydrophobic physical barrier [51].

The topic of obtaining NIPU composite materials has been actively studied. As conventional polyurethanes can be utilized for composite preparation, their non-isocyanate counterparts also fit this role successfully. There are three distinctive ways to incorporate NIPUs in composite materials. In the conventional approach, NIPUs are used as a polymer matrix and, thus, are modified with different compounds. The useage of nanofillers is especially promising, as even a low amount of nanofiller incorporated in NIPU matrix can improve its properties significantly. Examples include, but are not limited to, graphene oxide or POSS nanoparticles. The second way to incorporate NIPU in a composite material is to use it as an additive to another polymer matrix. The usage of NIPU as a filler improves mechanical properties of such polymer matrices as poly(propylene carbonate) or epoxy resin. The final approach to manufacturing NIPU composite materials is the synthesis of copolymers with NIPU as one of the building blocks. Epoxy compounds receive most attention in terms of copolymers for NIPUs. The copolymerization of non-isocyanate polyurethanes with epoxies allows for overcoming the flaws of the latter in terms of mechanical properties or chemical resistance. Taking into consideration the vast possibilities of combining different materials in the process of composite preparation, the progress in this field is far from over.

## 4. Processing of NIPU

As conventional polyurethanes, NIPUs can be further processed by various methods. Having similar or better thermal and mechanical properties than classical PUs, NIPUs can be extruded, injection-molded, or pressed, provided they show thermoplastic properties.

Magliozzi et al., investigated the benefits of the reactive extrusion process vs. PHU properties. Three different biscyclic carbonates (diglycerol dicarbonate, Seb-bCC-ester [bis-((2-oxo-1,3-dioxolan-4-yl)methyl)decanedioate], and Und-6DA-bisCC) with different reactivities were tested. Different diamine compounds were also used (1,4-diaminobutane (4DA), 1,6-diaminohexane (6DA), 1,10-diaminodecane (10DA), 12DA, m-xylylenediamine (xy-DA), and L-lysine). Depending on the composition, the polymerization process was performed at different temperatures, usually in the range of 80–95 °C, 120 °C, or 220 °C. After premixing the substrates (both manually and using a micro-compounder), they were injected into a twin-screw extruder. The initial rotation speed of 100 rpm was unchanged during the reaction. Parameters such as torque, true temperature, and pressure (ΔP) were continuously monitored. The PHUs obtained via reactive extrusion were compared with their analogs, which were synthesized through conventional bulk polymerizations. Kinetics and average molar masses, alongside the occurrence of side reactions, were evaluated. Certain compositions showed complete conversions of substrates in short reaction times (a few hours, where it could take whole days) and with no need for additional heating. Thanks to such specific circumstances and lowered reaction times, the creation of by-products was prevented almost completely. Additionally, the versatility of that approach concerning various monomer structures shows consistency, as *T_g_* and thermal properties of samples obtained with different diamines stay stable [52].

Fabrication of porous NIPU materials is also possible, as shown by the work of Cornille et al., who obtained cellular NIPUs by utilizing poly(methylhydrogenosiloxane) as a blowing agent. Polymer matrix was obtained in the reaction between selected carbonate compounds: poly(propylene oxide) bis-carbonate or trimethylolpropane tris-carbonate, and one of two amine compounds: ethylene glycol-based diamine or a derivative of C_18_ fatty acids diamine. 1,5,7-triazabicyclo[4.4.0]dec-5-ene (TBD) was used in the role of catalyst. Selected cyclo-carbonate compounds and the catalyst were put in the silicone mold and mixed with a mechanical stirrer for 3 min. The amine compound was mixed in afterward for around 3 min. When a homogenous mixture was obtained, the blowing agent was finally mixed in for 2 min. Foamed mixtures prepared in this manner were heated at 80 °C for 12 h, followed by the post-curing process at 120 °C for 4 h. NIPU foams underwent subsequent characterization of their structure. Apparent density measurements, as well as SEM and DMA analyses, were performed. The degree of cross-linking of the obtained foams was measured indirectly by the swelling index. Thermal properties were determined by TGA and DSC. The variation in functionality of cyclic carbonates and the composition of amine turned out to have a significant impact on the structure and thermal properties as well as the degree of cross-linking of the obtained foams. All these tests also displayed that the obtained foams were characterized by high apparent density and high flexibility. The apparent density of obtained non-isocyanate foams falls in the range of 194–295 kg/m^3^, which allows them to be classified as high-density foams. Cushion and shock adsorption properties of such materials show that NIPU foamed materials may find use in furniture or automotive industries, as well as fillers for shipment of packages [11].

Clark et al., conducted copolymerization of a sorbitol-derived bis-carbonate with hexamethylenediamine and cadaverine, which was obtained based on lysine without the need of utilizing any solvents. Analysis of the gases released during the foaming process established that the foaming agent was CO_2_ produced during polymerization. The foaming reaction was observed to happen right away upon heating mixed reactants. The reported self-foaming phenomenon showed a remarkable difference in the preparation route of foams compared to the “classical” addition of a foaming agent. Such an approach is also outstandingly efficient due to CO2 being confined within the pores rather than being released to the atmosphere. Moreover, the raw materials used to prepare this kind of NIPU foam are bio-based, inexpensive, and exhibit low toxicity. It was also stated that obtained materials might be useful in applications such as insulation or packaging [10].

**Scheme 18:**
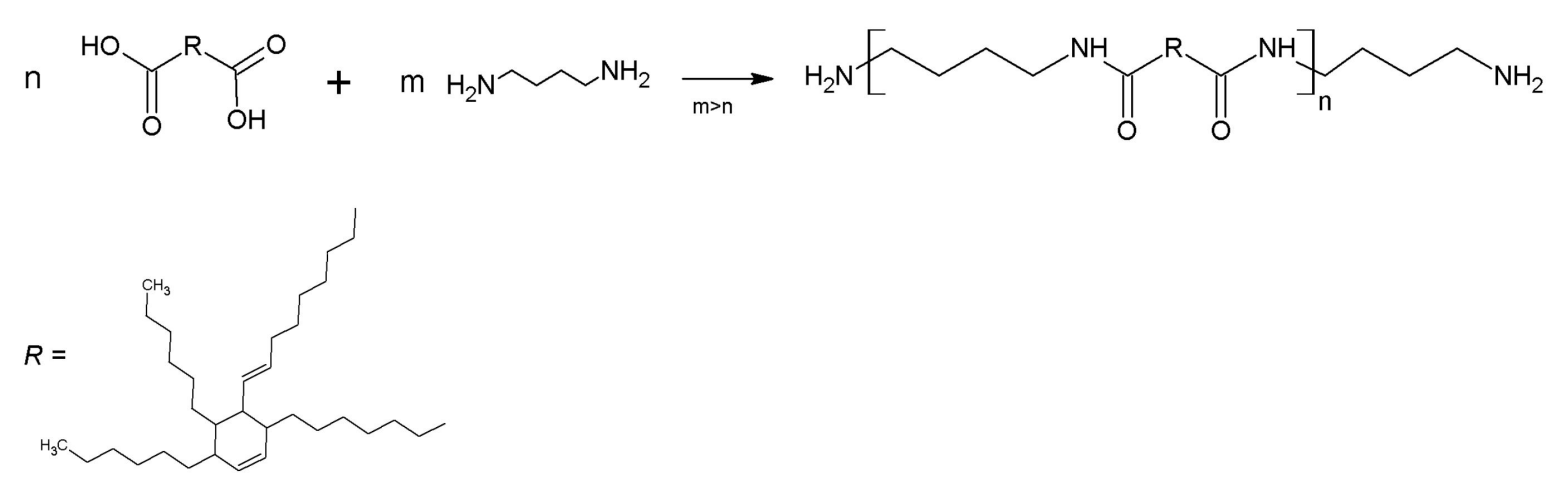
Synthesis of amino-end-terminated oligoamide [54].

Grignard et al., prepared NIPU foams utilizing the supercritical carbon dioxide (scCO_2_) foaming method. The matrices were based on poly(ethyleneglycol) biscyclocarbonate (PEG100) and two carbonated soybean oils (CSBO50, CSBO100) and amino telechelic oligoamide, which was obtained in the reaction between dimer fatty acid (Pripol 1013) and 1,4-butanediamine (Scheme 18) [53].

Foams were obtained by two methods: one- or two-step batch approaches. In the one-step method, CO_2_ was introduced in NIPU under supercritical conditions, where later depressurization occurred in a couple of seconds, resulting in the expansion of the sample. The two-step approach—developed posteriorly—showed better results in producing NIPU foams. The first step consisted of similar operations as in the one-step method, namely, saturating NIPU material with supercritical carbon dioxide (100 or 300 bar), while the second step was conducted by lowering the reactor temperature to 0 °C before depressurization. The foaming process took place after the depressurization was complete. It was carried out at elevated temperatures (80 °C or 100 °C). This approach yielded closed-cell NIPU foams with a cell diameter of 1–20 μm. Those samples were determined to have a density of circa 110 kg/m^3^ and thermal conductivity of 50 mW/mK, which is similar to glass wool or wood (45 and 55 mW/mK, respectively) [53]. Overall, this study showed that NIPU foams might be utilized as microcellular insulating materials [54].

Blattmann et al., prepared NIPU foams based on carbonated polyglycidylethers of TMP (TMPGC) and ethoxylated TMP (EO-TMPGC). Hexamethylene diamine (HMDA) was used as the second component of the matrices. Solkane 365/227 was utilized as a physical blowing agent. This liquid hydrofluorocarbon is reported to have no negative impact on the ozone layer in the atmosphere and to be non-flammable as well. Two carbonate compounds were mixed in a set ratio at 80 °C, and HMDA was added in the next step. The samples were then cured at 80 °C for 14 h, followed by a post-curing process for 4 h at 100 °C. For foam materials, TMPGC (60 wt%) and EO-TMPGC (40 wt%) were mixed in a similar manner as mentioned above but without any additional heating and with an addition of 1 wt% of 1,4-diazabicylco[2.2.2]octane (DABCO) as a catalyst. After the amine compound was mixed in, the composition was cooled down to room temperature (due to the exothermic nature of the reaction), when the blowing agent Solkane 365/227 was added into the mixture in the amount of 25 wt%. The sample was cured in the same way as non-foamed NIPUs. NIPU foam obtained in this manner was evaluated to have a density of 83 kg/m^3^ after being crushed. That result is stated to far surpass other in-development NIPU foams. Other properties, namely, hysteresis value of 13.3% and hardness of 3.0 kPa, were also superior compared to similar foamed NIPU products [11,55].

**Figure 5:**
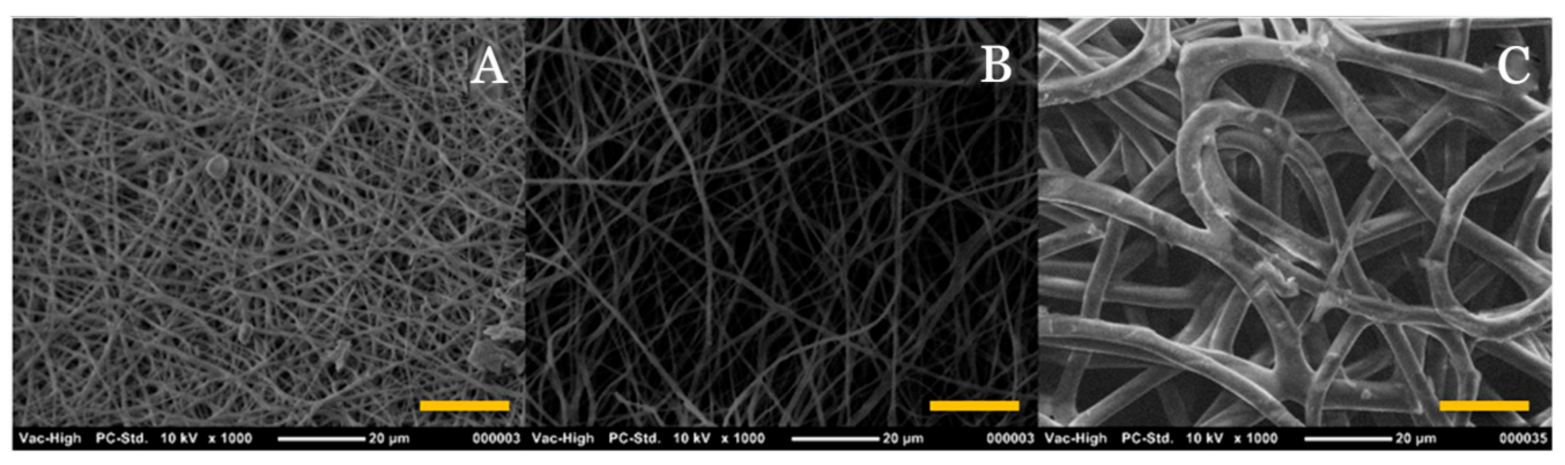
Scanning electron microscopy microscale images of (**A**) ES PA12HU-PTMO25; (**B**) ES PA12HU-PTMO33; (**C**) ES thermoplastic polyurethane (TPU) control fibers. Scale bars: 20 mm [56].

Electrospinning of plant oil-based non-isocyanate polyurethanes to produce fibrous materials for biomedical applications was conducted by Aduba and co-workers. Utilizing polymerization in the molten state of a plant oil-based cyclic-carbonate-bearing polyether soft segments and selected diamines, based on poly(tetramethylene oxide) (PTMO), yielded non-isocyanate, segmented poly(amide12 hydroxyurethane)s (PA12HUs). Using renewable, plant oil-based material and utilizing the electrospinning technique shines a new light on the fabrication of NIPU, while being in accordance with green chemistry postulates. The obtained samples were then dissolved in hexafluoro-2-propanol (HFIP) and electrospun (ES), resulting in materials shown in Figure 5 [56].

Electrospun fibrous substrates displayed nanoscale fiber morphology alongside uniformity in size distribution. The thermal analysis revealed that it had little effect on the thermal stability of NIPUs. Additionally, they exhibited similar mechanical properties to those of conventional thermoplastic polyurethanes (TPUs). Fibrous mats were characterized by elevated water uptake in comparison to TPU analogs. Cell viability and attachment assessment confirmed no cytotoxicity effects [56].

Digital free-form fabrication and rapid manufacturing conducted by 3D and 4D printing draws the attention of various industries and broadens the perspectives of polymer processing concerning customized additive manufacturing. Amidst additive manufacturing techniques, fused filament fabrication (FFF) or fused deposition modeling (FDM) are outstandingly solid with regard to additive manufacturing of thermoplastics. Usually, polymer filaments are introduced into the heated extrusion print head to ensure the 3D dispensing of the resulting polymer melts [57].

**Figure 6:**
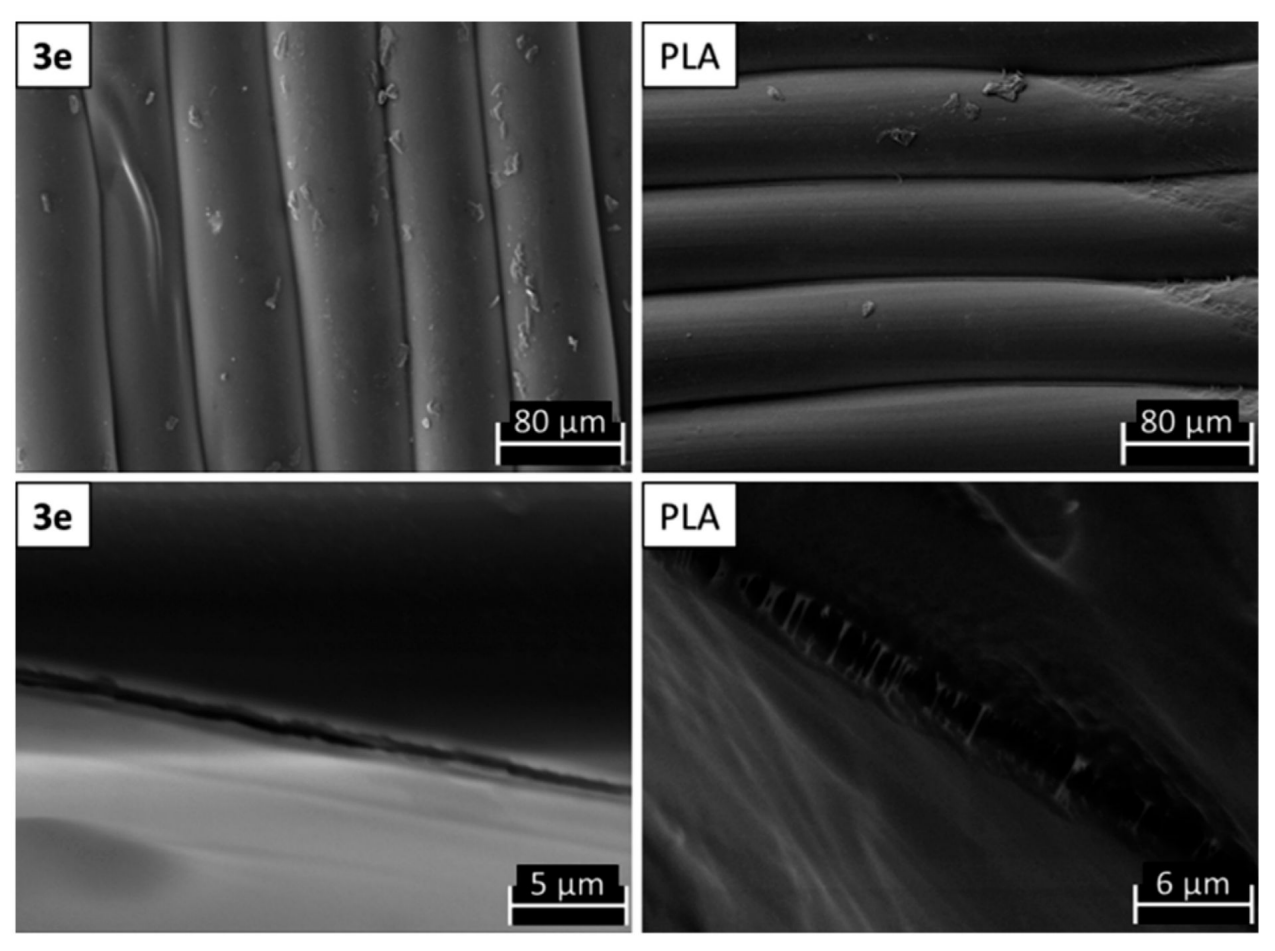
SEM imaging of 3D-printed dice made of PHU 3e (**above and below left**) and polylactide (PLA, **above and below right**) [58].

Schimpf et al., obtained semicrystalline polyhydroxurethane (PHU) thermoplastics via polyaddition of difunctional cyclic carbonates with 1,12-diaminododecane (DDA) using a twin-screw compounding extruder. Polyaddition reaction was conducted in the molten state. CCs were obtained by chemical fixation of CO_2_ with diepoxides. The process was conducted under an inert atmosphere (nitrogen) in the temperature range of 100–130 °C for up to 130 min. The main focus was to obtain profiles that would exhibit properties similar to those of traditional thermoplastic polyurethanes. Semicrystalline PHUs with tailored properties were obtained and used as filaments in extrusion-based additive manufacturing utilizing FFF or FDM techniques. Hydroxyl groups present in PHUs imply strong hydrogen bonding, which is beneficial with respect to the 3D printing technique. To maintain desired mechanical properties in 3D printing, suitable chain entwinement and adhesion between adjoining layers are necessary. It also prevents pore generation and reduces structural flaws. SEM images display the neighboring threads of material in a 3D-printed object—Figure 6. Polylactide (PLA) was used as a comparison to PHU (3e). While the build precision does not vary, strands in the PHU (3e) are regularly aligned near each other, enhancing the adhesion and improving interlayer adhesion and layer fusion [58].

It is possible to conduct the processing of non-isocyanate polyurethanes in the same or similar manner as conventional polyurethanes. Reactive extrusion was the favored method of choice for this process. Even though the reactivity of cyclic carbonates and amines may be lower than their counterparts in conventional PU synthesis—isocyanates and polyols—NIPUs may also be processed successfully via the reactive extrusion method. Listing further similarities between the processing methods for standard polyurethanes and NIPUs, the process of foaming deserves special attention. While water cannot be used as a chemical foaming agent for non-isocyanate polyurethanes, as there are no -NCO groups for water to react with, the foaming process of NIPUs can be executed by different means. Other methods, less widely used in industry, include, but are not limited to, electrospinning or 3D printing. Growing interest in NIPU materials shall accelerate development of their processing methods and their implementation on an industrial scale.

## 5. Conclusions

Isocyanates and polyols are commonly used in polyurethane synthesis. However, the development of new cleaner synthetic routes without the need to use diisocyanates gains increasing attention as it makes it possible to avoid the use of toxic and environmentally hazardous substrates. An ever-growing demand for harmless, environmentally friendly chemicals accelerates the search for new NIPUs, especially those synthesized via the polyaddition reaction of cyclic carbonates and diamines, yielding poly(hydroxy urethane)s. Other useful pathways include transurethanization (polycondensation), ring-opening polymerization, and rearrangement reactions. Numerous cyclic carbonates with different functional groups can be obtained, and design of the molecular structure of cyclic carbonates and their oligomers is crucial for the performance of final products. Favorable bio-based feedstocks, e.g., derivatives of plant oils, could be successfully used as precursors in cyclic carbonate synthesis. Polyamines used for the aminolysis of cyclic carbonates may also be synthesized on the basis of bio-based precursors. The scope of possible applications for these compounds from renewable resources is certainly going to be expanded due to the growing interest of industry and academia. The elaboration of novel isocyanate-free and phosgene-free ways of polyurethane synthesis and the use of novel renewable resources, including carbon dioxide, are considered as major challenges in new material development.

In the field of composites and copolymer materials, NIPUs show promising possibilities. Ease of chemical modification due to the presence of hydroxyl side groups, as well as the thermal and mechanical performance often surpassing those of conventional Pus, are qualities acknowledged in modern materials science and technology. As stated in this review, NIPUs may be utilized in composite materials in many different ways. The most common approach, with NIPU acting as a polymer matrix, attracted the vast majority of attention. Fillers used in those composite materials may be introduced as blends so that only physical interactions occur between them and the NIPU matrix. Although this method ensures substantial improvements of mechanical and thermal properties of final products, the possibility to chemically bind used fillers with NIPU matrices is what plays a significant role in the field of non-isocyanate polyurethane composites. Furthermore, both organic and inorganic fillers can be modified prior to their introduction into NIPU matrix possessing reactive groups, such as carbonate, amine, or hydroxyl groups. It was shown that chemically bound fillers or nanofillers enhance the final properties of NIPU composites to a greater extent than their non-reactive counterparts. Aiming at possible applications in insulating, automotive, furniture, or packaging industries as well as anti-corrosive coatings, NIPU matrices are modified to display low thermal conductivity yet high resistance, outstanding resistance to various chemical agents, and hydrolysis, alongside extraordinary mechanical properties—high Young’s modulus, elongation at break, and tensile strength. NIPUs can be utilized not only as polymer matrices but also as additives or (co)polymers; e.g., they were found to improve the thermal properties of epoxy resins.

The ability to be processed by well-known classical techniques, such as extrusion and injection molding, as well as by electrospinning and 3D printing, opens a broad spectrum of future applications for NIPU materials in different fields, such as in the biomedical sector. Addressing one of the drawbacks of non-isocyanate polyurethanes—low reactivity of some amine-carbonate systems—the reactive technique seems to be an advantageous processing method. It was also shown that NIPU polymers and composites may also be obtained as porous materials or scaffolds, which is again desirable for biomedical applications.

Although NIPUs draw increasing attention from modern industry and academia, which is reflected by the number of emerging papers and patent applications on the matter, it is obvious that a lot of phenomena regarding NIPUs still remain unsolved. Concentrated efforts must be undertaken to efficiently tackle those issues, and a dedicated review work shall help to show recent research trends on these promising materials.

## Figures and Tables

**Table 1:** Effect of methanol and the introduction mode of reactants on NIPU prepolymer characteristics [25].

	*N* (PPO-5CC):*n* (EDA)	Addition Method	Alcohol Type	Alcohol Amount (wt%)	*M_n_* (g·mol^−^^1^)	*M_w_* (g·mol^−^^1^)	Polymer Dispersity Index	Prepolymer Viscosity at 70 °C (Pa·s)
NIPU-1	1:2	All-in method	-	-	2400	3470	1.57	1.51
NIPU-2	1:2	All-in method	Methanol	50	3660	6510	1.78	2.01
NIPU-3	1:2	Reverse method	Methanol	50	1840	3460	1.88	1.57
NIPU-4	1:2	Forward method	Methanol	50	4300	7050	1.64	2.71

## Data Availability

Data sharing not applicable.
