# Correction: Stachak et al. Recent Advances in Fabrication of Non-Isocyanate Polyurethane-Based Composite Materials. *Materials* 2021, *14*, 3497

**DOI:** 10.3390/ma18122767

**Published:** 2025-06-12

**Authors:** Piotr Stachak, Izabela Łukaszewska, Edyta Hebda, Krzysztof Pielichowski

**Affiliations:** Department of Chemistry and Technology of Polymers, Faculty of Chemical Engineering and Technology, Cracow University of Technology, Warszawska 24, 31-155 Cracow, Poland; izabela.lukaszewska@doktorant.pk.edu.pl (I.Ł.); edyta.hebda@pk.edu.pl (E.H.); kpielich@pk.edu.pl (K.P.)

In the original publication [1], reference 25 is incorrect. The corrected reference 25 has now been updated and should read as follows:

25. Ke, J.; Li, X.; Jiang, S.; Liang, C.; Wang, J.; Kang, M.; Li, Q.; Zhao, Y. Promising approaches to improve the performances of hybrid non-isocyanate polyurethane. *Polym. Int.* **2019**, *68*, 651–660.

In addition, since the original email address of the corresponding author, Piotr Stachak, is no longer active, it should be updated to the following: “stachak.pe@gmail.com”.

The authors state that the scientific conclusions are unaffected. This correction was approved by the Academic Editor. The original publication has also been updated.
